# SuFEx‐Enabled Reprogramming of Flavonoids for Selective α‐Glucosidase Covalent Inhibition

**DOI:** 10.1002/advs.75869

**Published:** 2026-05-29

**Authors:** Fengyu Guo, Liwei Zhang, Minlong Wang, Yanjin Du, Tianyu Zhang, Hao Chen, Jiazeng Sun, Yang Yu, Zude He, Jie An, Xiaoxu Zhang, Weilin Lin, Fazheng Ren, Pengjie Wang, Ping Liu

**Affiliations:** ^1^ Department of Nutrition and Health China Agricultural University Beijing China; ^2^ Guangzhou Women and Children's Medical Center GMU‐GIBH Joint School of Life Sciences Guangzhou Medical University Guangzhou China; ^3^ National Key Laboratory of Immunity and Inflammation Suzhou Institute of Systems Medicine Institution Chinese Academy of Medical Science & Peking Union Medical College Suzhou China

**Keywords:** SuFEx click chemistry, covalent inhibitors, flavonoid reprogramming, α‐glucosidase selectivity, metabolic regulation

## Abstract

Selective inhibition of intestinal α‐glucosidase is an established strategy for controlling postprandial hyperglycemia. However, currently available inhibitors are predominantly reversible and nonselective, leading to incomplete target engagement, concomitant α‐amylase inhibition, and gastrointestinal side effects associated with gut microbiota perturbation. Here, we report a SuFEx‐enabled reprogramming strategy that converts natural flavonoids into highly selective α‐glucosidase covalent inhibitors. Guided by the structure of human intestinal maltase–glucoamylase, fluorosulfate warheads were introduced via Sulfur(VI) Fluoride Exchange (SuFEx) click chemistry, yielding covalently active derivatives with markedly enhanced affinity and selectivity toward α‐glucosidase over α‐amylase. The lead compound FS6‐2 forms an active‐site‐directed covalent interaction with a conserved lysine residue, resulting in sustained inhibition of starch digestion in vitro and in vivo. In diabetic mice, SuFEx‐modified flavonoids improved glycemic control, preserved pancreatic integrity, and remodeled gut microbiota composition, restoring microbial diversity and metabolic function. This work validates SuFEx‐enabled covalent reprogramming as a powerful strategy to transform dietary natural products into precision metabolic medicines, offering a versatile platform for developing selective, microbiota‐compatible metabolic therapeutics.

## Graphical abstract

Selective inhibition of intestinal α‐glucosidase remains limited by poor enzyme specificity and off‐target metabolic effects. Here, SuFEx click chemistry is used to reprogram natural flavonoids into covalent inhibitors with enhanced α‐glucosidase selectivity and controlled reactivity. This strategy enables effective regulation of carbohydrate digestion and systemic metabolic homeostasis while minimizing gastrointestinal disturbance.

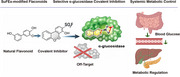

## 1 Introduction

Excessive carbohydrate consumption is a major driver of type II diabetes worldwide, and chronic hyperglycemia contributes to inflammation, impaired lipid metabolism, and other metabolic complications [1, 2]. One well‐established therapeutic approach involves inhibiting α‐glucosidase, the key enzyme responsible for the final steps of carbohydrate digestion, thereby slowing glucose absorption and mitigating postprandial hyperglycemia [3]. Clinically approved inhibitors—acarbose, miglitol, and voglibose—validate this strategy, yet their simultaneous inhibition of α‐glucosidase and amylase in the small intestine results in substantial amounts of undigested polysaccharides reaching the colon [4]. This can disrupt gut microbial homeostasis and cause gastrointestinal side effects such as bloating and diarrhea. Therefore, developing selective α‐glucosidase inhibitors that minimize off‐target carbohydrate digestion represents a promising direction for safer and more effective glycemic control [5, 6].

To improve both selectivity and pharmacological performance, covalent modification has gained considerable attention as a powerful molecular design strategy [7]. Unlike reversible inhibitors, covalent inhibitors form irreversible or long‐lasting bonds with nucleophilic residues in enzyme active sites, producing sustained target engagement and heightened potency [8, 9]. This approach has been successfully applied to several clinically important enzymes, including Bruton's tyrosine kinase and epidermal growth factor receptor [10, 11]. A central challenge in covalent inhibitor design is the careful selection of the electrophilic warhead, which governs target specificity, reaction kinetics, and overall therapeutic window [12, 13]. Advances in bioorthogonal chemistry have greatly expanded the repertoire of tunable warheads available for covalent drug design, enabling more precise, and controlled protein modification [14, 15].

Among these advances, Sulfur(VI) Fluoride Exchange (SuFEx) click chemistry has emerged as a versatile and highly attractive platform for the modification of bioactive molecules. Although the SuFEx linker family has rapidly expanded to include a broad range of connective hubs, such as sulfonyl fluorides (─SO_2_F), sulfamoyl fluorides (‐NHSO_2_F), and sulfondiimidoyl fluorides [16], we deliberately selected the aryl fluorosulfate (‐OSO_2_F) warhead for the present study. In contrast to conventional highly reactive electrophiles or more hydrolysis‐prone SuFEx linkers, aryl fluorosulfates exhibit tunable “Sleeping Beauty” reactivity, remaining essentially inert until activated by a specific protein microenvironment [17]. This unique property enables SuFEx‐derived inhibitors to evade nonspecific reactions while minimizing off‐target engagement. Accordingly, the ‐OSO_2_F moiety provides a robust structural foundation for the design of covalent inhibitors that achieve an effective balance between reactivity, potency, and selectivity [18].

In the study for selective α‐glucosidase inhibitors capable of modulating glucose metabolism, numerous chemical classes have been explored, including newly synthesized small molecules, repurposed drugs, and natural products [19]. Among them, flavonoids stand out due to their abundance in the human diet and their multifaceted metabolic benefits, including attenuation of hyperglycemia [20]. However, because highly active flavonoids often interact with both α‐amylase and α‐glucosidase, they typically lack sufficient selectivity toward α‐glucosidase, which limits their utility as targeted therapeutics [18, 19, 21]. This arises from distinct structure–activity relationships: α‐amylase inhibition is associated with the C2═C3 double bond and hydroxyl groups at A5, B3, and B4, whereas α‐glucosidase inhibition correlates with hydroxylation at B3, B4, and C3. This challenge highlights the need for strategies—such as covalent SuFEx‐based modification—to refine flavonoid scaffolds and transform them into more selective and potent α‐glucosidase inhibitors.

**FIGURE 1:**
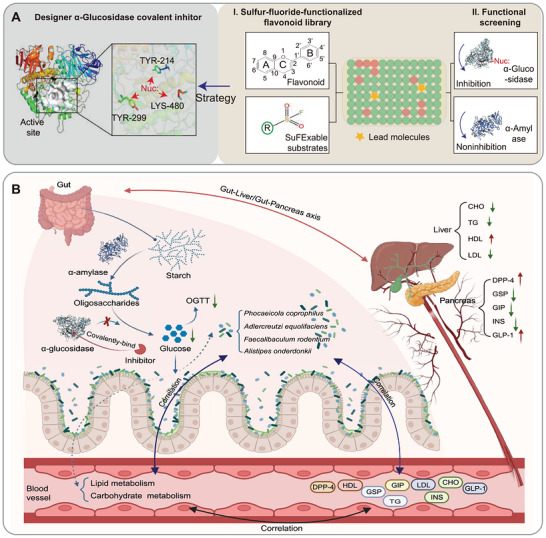
(A) Derivatization screening process of arylfluorosulfate derivatives and the covalent binding sites of representative derivatives with α‐glucosidase. (B) Mechanism intentions of arylfluorosulfate derivatives in vivo after intervention.

Herein, we report a SuFEx‐enabled covalent reprogramming strategy that modified flavonoids into highly selective α‐glucosidase inhibitors with systemic metabolic benefits (Figure 1). By introducing fluorosulfate warheads through SuFEx click chemistry, we generated a series of covalently active flavonoid derivatives that exhibit markedly enhanced affinity and selectivity toward α‐glucosidase over α‐amylase. Mechanistic analyses reveal that this selectivity arises from active‐site‐directed both covalent and non‐covalent engagement. The lead compounds effectively inhibited starch digestion in vitro and in vivo, resulting in sustained glycemic control and improved pancreatic integrity in diabetic mice. Beyond enzymatic inhibition, multi‐omics analyses demonstrated that SuFEx‐modified flavonoids remodel gut microbiota composition, restore microbial diversity, and modulate key metabolic pathways linked to glucose and lipid homeostasis. Together, this work establishes SuFEx chemistry as a powerful platform for covalent functionalization of natural products, offering a rational route to selective enzyme targeting and microbiota‐informed metabolic regulation.

## 2 Results

### 2.1 Rational Design and In Vitro Identification of Covalent α‐Glucosidase Inhibitors

Structural analysis of human intestinal maltase–glucoamylase (PDB: 2QMJ) reveals a well‐defined catalytic pocket enriched in reactive residues (e.g., Lys and Tyr), providing a strong substrate for selecting fluorosulfate (R–OSO_2_F) warheads for the rational design of covalent enzyme inhibitors [22]. Given that flavones and chalcones possess multiple modifiable sites and potential glucose metabolism‐regulating properties, 11 compounds (F1–F11) were selected for further titrated modification with arylfluorosulfate via SuFEx click chemistry (Table S1).

**FIGURE 2:**
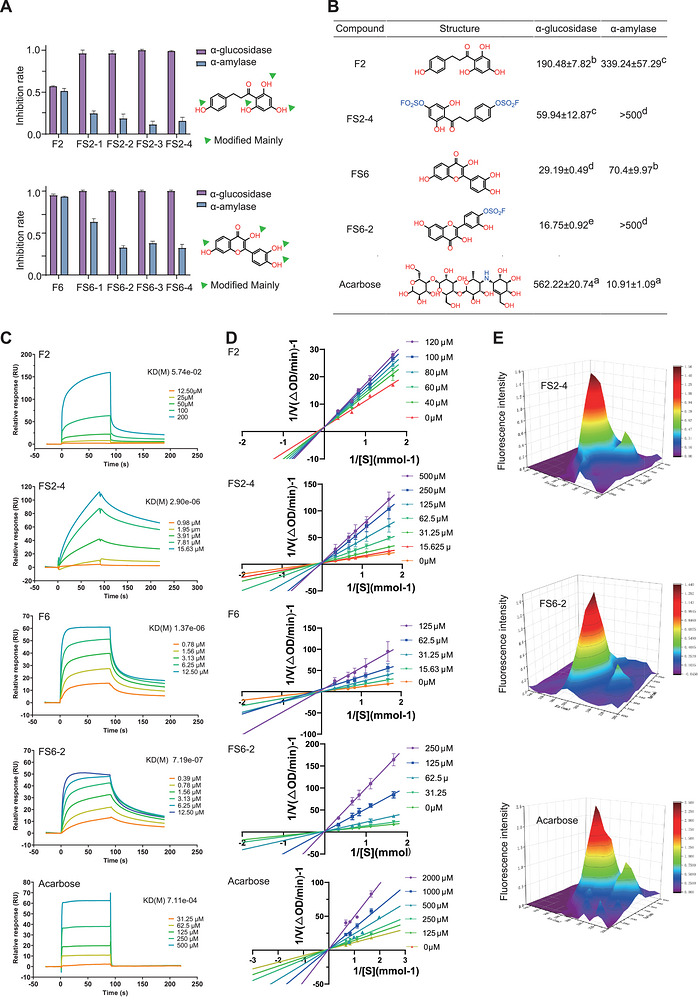
Enzyme inhibitory activity of arylfluorosulfate derivatives. (A) Enzyme inhibitory activity of representative sulfonyl fluoride derivatives. (B) Inhibitory activities against α‐glucosidase and α‐amylase of inhibitors (IC_50_, µm)^f^, ^f^ Each IC_50_ value is the mean ± SEM from at least three independent experiments. The different superscripts in the same column mean significant differences (P < 0.05). (C) SPR results of α‐glucosidase by inhibitors F2, FS2‐4, F6, FS6‐2, and Acarbose. (D) Lineweaver‐Burk diagram of inhibition of α‐glucosidase by inhibitors. Different concentrations of inhibitors are listed in their respective legend entries. The illustration is a quadratic plot of the slope and [*I*] from the Lineweaver‐Burk plot. (E) Synchronous and 3D fluorescence of the enzyme under the influence of compounds.

To identify lead α‐glucosidase inhibitors, we synthesized a library of ∼45 flavonoid arylfluorosulfate derivatives (FSn) and evaluated their inhibitory activity against α‐glucosidase and α‐amylase in vitro. While FS1, FS3, FS5, F7, F8, and F9 exhibited a modest increase in α‐glucosidase selectivity, the 3‐substituted derivatives of F4 showed reduced selectivity. Notably, all modified compounds except F4 demonstrated improved α‐glucosidase selectivity compared to their parent structures (Figure S1), suggesting that the ‐OSO_2_F moiety enhances target specificity. Among these, F2 and F6 derivatives displayed the most significant improvement in both selectivity and inhibitory activity upon ‐OSO_2_F incorporation. Thus, F2, F6, and their derivatives were selected for isolation, purification, and structural verification via NMR and high‐resolution mass spectrometry (HR‐MS) (Figure 2A and Figures S13–S19). The purity of the lead candidate FS6‐2 was determined to be 92% by HPLC analysis (Figure S21), which is appropriate for subsequent pharmacological evaluations.

The in vitro enzyme inhibition assay determined the IC_50_ values of arylfluorosulfate derivatives against α‐glucosidase and α‐amylase (Figure 2B). As illustrated in Figure 2B, the IC_50_ of acarbose for α‐glucosidase and α‐amylase was 562.22 ± 20.74 and 10.91 ± 1.09 µm, respectively. Notably, lower IC_50_ values indicate stronger enzyme inhibitory potency. Derivatives FS2‐4 and FS6‐2 exhibited significantly enhanced α‐glucosidase inhibition compared to their parent compounds, with IC_50_ values of 59.94 ± 7.82 and 16.75 ± 0.92 µm, respectively. Furthermore, the introduction of ‐OSO_2_F groups markedly improved the selectivity of these derivatives toward α‐glucosidase over α‐amylase. Parent compounds F2 and F6 already demonstrated strong α‐glucosidase selectivity (IC_50_ values: 190.48 ± 7.82 and 29.19 ± 0.49 µm), outperforming acarbose (562.22 ± 20.74 µm). However, the derived compounds FS2‐4 and FS6‐2 achieved even greater selectivity and inhibitory activity, highlighting the potential of structural modification to optimize therapeutic candidates. These results prompted a quantitative investigation of binding affinity and selectivity.

### 2.2 SuFEx Modification Enhances the Affinity and Selectivity Toward a‐Glucosidase

To further investigate the binding mechanisms of the flavonoids and their derivatives, we first quantified the affinities of the lead inhibitors to α‐Glucosidase using surface plasmon resonance (SPR) (Figure 2C). The SPR experiments revealed that the derivatized compounds exhibited enhanced binding affinity to the enzyme, with KD values of FS2‐4 (2.90 × 10^−^^6^ m) and FS6‐2 (7.19 × 10^−^^7^ m), which were significantly lower than those of the parent compounds (KD_F2_ = 5.72 × 10^−^^2^, KD_F4_ = 1.37 × 10^−^^6^ m) and substantially lower than that of the positive control drug, acarbose (KD_Acarbose_ = 7.11 × 10^−^^4^ m). Notably, the sub‐micromolar binding affinity of FS6‐2 to α‐Glucosidase highlights its potential for further investigation.

We also evaluated the detailed enzyme kinetics to accurately reproduce the inhibitory activity of enzyme inhibitors. We further characterized the enzyme inhibition kinetics to elucidate the mechanism of action of the inhibitors. Inhibition type, maximum reaction velocity (*V_max_*), and Michaelis constant (*K_m_*) were analyzed via Lineweaver‐Burk plots. As depicted in Figure 2D, the linear fits converge on the *y*‐axis, with increasing inhibitor concentrations causing a reduction in the *x*‐axis intercept (1/*K_m_*), an increase in slope (*K_m_*/*V_max_*), and no change to the *y*‐axis intercept (1/*V_max_*). This kinetic profile indicates constant *V_m_* and decreasing *K_m_*, consistent with competitive inhibition where the inhibitor competes with the substrate for the enzyme's active site. Binding affinity was quantified using Equations (3) and (4) to calculate competitive (*K_ic_*) and uncompetitive (*K_iu_*) inhibition constants (Table S2). Derivatives FS2‐4 and FS6‐2 exhibited *K_ic_* values of 53.67 ± 11.57 and 6.04 ± 2.87 µm, respectively, demonstrating significantly stronger binding to α‐glucosidase than their parent compounds. This enhanced affinity, driven by the introduction of the ‐OSO_2_F group, correlates with their reduced IC_50_ values, underscoring the critical role of this structural modification in improving inhibitory potency. These observations validate that the SuFEx modification dramatically enhances the non‐covalent anchoring of the inhibitor within the enzyme's active site.

To further validate binding interactions and thermodynamics, we conducted fluorescence quenching experiments. α‐Glucosidase's intrinsic fluorescence (Trp/Tyr residues) reflects enzyme concentration. Stern‐Volmer analysis at three temperatures showed redshifted emission wavelengths (*λ_em_*) in inhibitor‐bound spectra, indicating partial structural unfolding from microenvironmental changes, and exposed residues (Figure S2). As shown in Figure S3, the linear concentration‐dependent fluorescence quenching of α‐glucosidase suggests a static quenching mechanism mediated by inhibitor binding [23, 24]. This is supported by *K_q_* values exceeding the dynamic quenching limit (2.0 × 10^10^ L/mol·s; Table S3). The Stern‐Volmer constants (*K_sv_*) followed the trend FS6‐2 > F6 > FS2‐4 > F2 > Acarbose, indicating enhanced binding affinity in derived compounds (Table S3). The near‐unity *n* values confirm 1:1 stoichiometry, while *K_a_* values (∼10^4^/M for FS2‐4/FS6‐2) demonstrate strong enzyme affinity. Notably, the ‐OSO_2_F group in derivatives likely augments interactions beyond the parent flavonoids' phenolic hydroxyl‐mediated binding. The temperature‐dependent *K_a_* decrease (Table S3) further reveals an exothermic, enthalpy‐driven process. The thermodynamic parameters (ΔH, ΔS, and ΔG) calculated from Formula (Table S4) provide further insight into the binding mechanism. The negative ΔH values confirm an exothermic process consistent with the observed temperature‐dependent decrease in *K_a_*. The negative ΔH and ΔS values suggest that van der Waals forces and hydrogen bonds dominate the interaction, while hydrophobic and electrostatic effects play minor roles. This aligns with previous findings for flavonoid‐α‐glucosidase complexes, where hydrogen bonding and hydrophobic interactions were key stabilizing forces.

To further reveal structural/conformational rearrangements, synchronous and 3D fluorescence were generated. Synchronous fluorescence spectroscopy was used to record the microenvironment changes of α‐glucosidase after the addition of inhibitors, and the results are shown in Figure 2E. It can be seen from the figure that with the increase of inhibitor concentration, the synchronous fluorescence intensity of α‐glucosidase at Δλ = 15 and 60 nm gradually decreases. The results suggest that the binding of the inhibitor to α‐glucosidase exposes the chromophore to more aqueous environments, decreasing fluorescence intensity. Notably, with the increase of the concentration of F2, F6, and FS6‐2 inhibitors, the fluorescence peak of Tyr residue had a slight blueshift, indicating that the hydrophobicity of the Tyr residue microenvironment was slightly enhanced. Under the inhibition of FS2‐4, the fluorescence peak of Tyr residue showed a significant redshift (303–317 nm), indicating that the introduction of ‐OSO_2_F group changed the interaction between parent compound F2 and Tyr residue, and enhanced the polarity of the microenvironment of Tyr residue. At the same time, only under F2 and FS2‐4 conditions, the fluorescence peaks of Trp residues showed a significant redshift (340 and 340–345nm), indicating that the polarity of the microenvironment of Trp residues was enhanced. Under F6 and FS6‐2 conditions, the maximum fluorescence peak of Trp did not move, indicating that the introduction of ‐OSO_2_F group did not change the interaction of the parent compound F6 on Trp residues. Figure S3 presents the RSQF values of the F2 compound at Δλ = 15 and 60 nm, which are nearly equal, indicating that the contribution of Trp and Tyr residues to α‐glucosidase fluorescence quenching is almost equal. For F2, F6, and FS6‐2, at the same compound concentration, the RSFQ value of Trp (Δλ = 60 nm) was significantly higher than that of Tyr (Δλ = 15 nm), indicating that Trp contributed more to intrinsic fluorescence quenching, and the binding site of the compound on α‐glucosidase was closer to Trp than that of Tyr residue.

### 2.3 Proximity‐Driven Covalent Engagement Dictates α‐Glucosidase Selectivity

**FIGURE 3:**
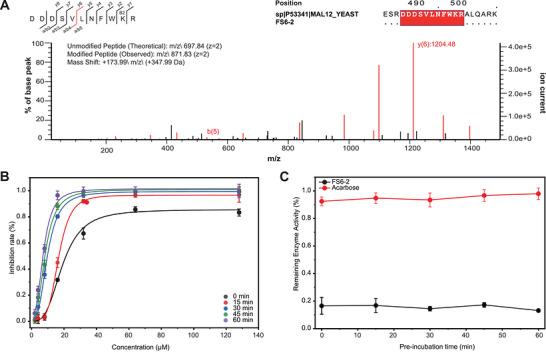
Mechanistic validation of FS6‐2 as a targeted covalent inhibitor of α‐glucosidase. (A) High‐resolution LC‐MS/MS peptide mapping spectrum confirming the precise covalent modification of the enzyme by FS6‐2. (B) Time‐dependent inhibition profiles of FS6‐2 against α‐glucosidase. (C) Jump‐dilution reversibility assay evaluating the recovery of enzymatic activity.

The interaction between lead compounds and their target protein is a key determinant of inhibitory efficacy. To elucidate the molecular basis of their high affinity, we employed LC‐MS/MS peptide mapping to investigate whether the ‐OSO_2_F warheads on our lead candidates (FS2‐4 and FS6‐2) successfully executed covalent conjugation. Intriguingly, LC‐MS analysis validated the covalent binding of only FS6‐2 to the target protein (Figure 3A). The results revealed a mass shift consistent with the formation of an ‐OSO_2_F adduct, which provided evidence for the covalent bond formation exclusively between FS6‐2 and Lys480 of α‐glucosidase [18]. In stark contrast, no covalent binding was observed for FS2‐4 under the same experimental conditions. To assess the critical role of Lys480, we evaluated the affinity between FS6‐2 and a mutant α‐glucosidase where Lys480 was replaced by Gly480. The binding affinity dropped dramatically from the sub‐micromolar to the sub‐millimolar range, highlighting the critical role of the covalent interaction between Lys480 and FS6‐2 in stabilizing enzyme–ligand complexes (Figure S4A).

While the thermodynamic and fluorescence analyses described above established a strong initial reversible interaction, the markedly greater inhibitory potency of FS6‐2 relative to its parent compounds and acarbose, together with the deliberate incorporation of a reactive SuFEx warhead, strongly suggested the occurrence of a subsequent irreversible covalent binding event [8]. Having identified FS6‐2 as the lead targeted covalent inhibitor, we therefore focused subsequent standard enzyme kinetic analyses exclusively on this compound to further validate its two‐step covalent mechanism. In the time‐dependent inhibition assay, the IC_50_ of FS6‐2 against α‐glucosidase showed a pronounced and progressive leftward shift as the pre‐incubation time increased from 0 to 60 min (Figure 3B). Specifically, the IC_50_ decreased from approximately 19.03 µm at 0 min to 7.40 µm at 60 min, consistent with the time‐dependent inactivation behavior characteristic of covalent inhibitors [25]. In addition, a jump‐dilution assay was performed to evaluate binding reversibility [26]. Following 100‐fold dilution of the preformed enzyme–inhibitor complex, the clinically used reversible inhibitor acarbose exhibited near‐complete recovery of enzymatic activity at all examined time points. By contrast, FS6‐2 induced a profound loss of enzyme activity, with no significant recovery observed even 60 min after dilution (Figure 3C). Collectively, these kinetic results provide compelling evidence that FS6‐2 acts as a targeted covalent inhibitor of α‐glucosidase [27].

**FIGURE 4:**
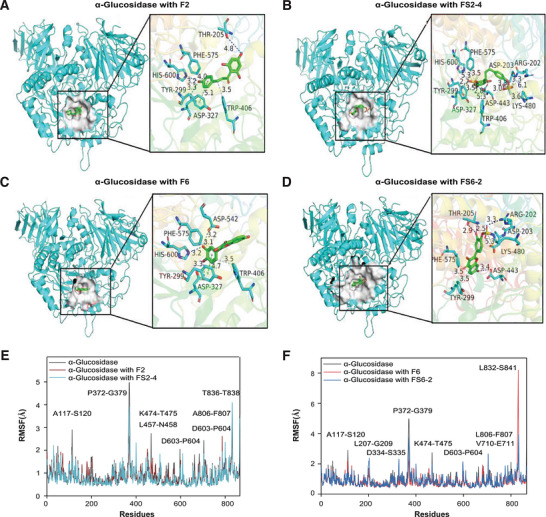
Mechanism of enzyme inhibition by fluorosulfate derivatives. (A–D) Molecular docking of α‐glucosidase (PDB: 2QMJ) with F2 (A), FS2‐4 (B), F6 (C), and FS6‐2 (D). The dashed blue lines represent hydrogen bonds, the dashed gray lines represent hydrophobic interactions, the dashed green lines represent *π*–*π* stacking, and the dashed yellow lines represent salt bridge (ionic bond). (E) present the RMSF values of binding site residues in both free proteins, α‐glucosidase with F2 complex systems, and α‐glucosidase with F2‐4 complex systems. (F) present the RMSF values of binding site residues in both free proteins, α‐glucosidase with F6 complex systems, and α‐glucosidase with F6‐2 complex systems.

Comparative molecular docking and molecular dynamics simulations were conducted to clarify why the structural analogue FS2‐4, despite its high binding affinity, failed to form a covalent bond. Using the crystal structure of α‐glucosidase (PDB: 2QMJ), we performed computational docking, revealing that all five tested molecules predominantly occupy the catalytic active sites at the −1 and +1 subsites. Like acarbose, compounds F2 and F6 primarily engage the active site through multiple hydrogen bonds (Figure 4A,C and Figure S5P). However, the binding modes and spatial orientations of the SuFEx‐modified derivatives diverge significantly. While FS2‐4 forms an additional hydrogen bond with the catalytic nucleophile Asp443 at the −1 subsite (Figure 4B), its ‐OSO_2_F warhead is oriented away from Lys480, precluding covalent engagement. Conversely, the binding mode of FS6‐2 markedly differs from its parent compound F6 (Figure 4D). While the flavonoid core of F6 occupies the ‐1 subsite, FS6‐2 replaces this moiety with the ‐OSO_2_F group. Although the hydrogen bond with Asp542 is retained, additional interactions arise with the catalytic nucleophile Asp443 and a salt bridge with Arg526. These modifications stabilize FS6‐2 at the ‐1 subsite by strengthening electrostatic interactions. Further stabilization is achieved through hydrogen bonds with Phe450 and Lys480. Most importantly, the ‐OSO_2_F group of FS6‐2 was positioned within reactive distance of the Lys480 nucleophile, thereby enabling the covalent bond subsequently confirmed by MS/MS analysis. This clear divergence highlights the proximity‐driven and “Sleeping Beauty” characteristics of SuFEx chemistry: the warhead remains functionally inert even within the target pocket unless the molecular scaffold precisely orients it toward a compatible nucleophilic residue. In addition, docking analysis against α‐amylase suggested that derivatization weakened compound–enzyme interactions, further supporting the observed selectivity profile (Figure S6).

Molecular dynamics simulations further supported the predicted binding orientations. All systems except α‐glucosidase‐FS6‐2 showed stable root‐mean‐square deviation (RMSD) (∼1.0 Å), while FS6‐2's flexibility caused higher deviations (∼1.3 Å vs. parent compound F6; Figure S5A,F,K). Most residues exhibited root‐mean‐square fluctuation (RMSF) <2 Å, with fluctuations distal to the active site, indicating minimal structural disruption, consistent with acarbose‐bound α‐glucosidase studies (Figure 4E,F and Figure S5L) [28]. Energy decomposition identified critical residues: For F2, ASP337, ILE364, TYR299, MET444, TRP406, GLU446, ARG526, GLY541, PHE575, and HIS600 favored binding, while ASP443 hindered it (Figure S5D). FS2‐4 strengthened interactions with ASP443/542 and other residues (Figure S5E). F6 relied on ASP203, TYR299, ASP327, TRP406, MET444, PHE450, LYS480, ASP542, PHE575, and HIS600 (Figure S5I). FS6‐2 introduced new bonds with ASP443/PRO407 and enhanced TRP406/ASP542/PHE575 interactions (Figure S5J). Asp443, Arg526, and Asp542 played critical roles in stabilizing the ‐OSO_2_F‐modified compounds through hydrogen‐bonding and salt‐bridge interactions, consistent with the docking results. These residues collectively strengthened inhibitor–enzyme binding and helped position the SuFEx warhead in a productive orientation. Taken together, these findings identify SuFEx‐enabled covalent anchoring as the key structural basis for the selective inhibition of α‐glucosidase.

Furthermore, given that the aryl fluorosulfate warhead could in principle react with other nucleophilic residues, we extended our selectivity evaluation to a broader panel of key gastrointestinal digestive enzymes [7]. Even at a high concentration of 200 µm, FS6‐2 showed negligible inhibitory activity against pepsin, trypsin, and pancreatic lipase, with relative enzyme activities remaining close to 100% and comparable to those observed for the clinical drug acarbose (Figure S7). In contrast, the corresponding positive controls—Pepstatin A, PMSF, and Orlistat—completely abolished enzymatic activity under the same assay conditions, thereby confirming the sensitivity and reliability of the assay system [17].

These results provide strong evidence that the ‐OSO_2_F warhead does not indiscriminately react with nucleophilic proteins in the complex, pH‐variable gastrointestinal environment. Instead, its covalent reactivity appears to be strictly proximity‐driven and context‐dependent, becoming activated only when the flavonoid scaffold precisely positions the warhead within the unique active‐site pocket of α‐glucosidase. This high degree of target specificity helps mitigate concerns regarding off‐target covalent toxicity and supports the favorable safety profile of FS6‐2 for oral administration.

### 2.4 SuFEx‐Modified Flavonoids Inhibit Starch Digestion In Vitro and In Vivo

**FIGURE 5:**
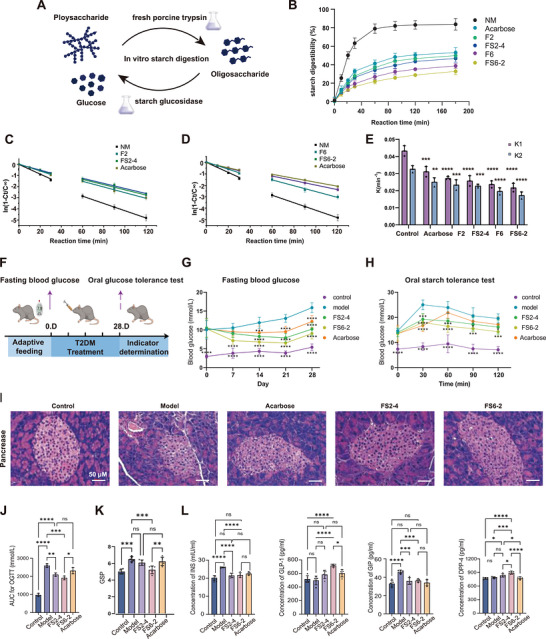
Post‐intervention recovery of T2DM‐related indicators. (A) Schematic diagram of in vitro starch digestion experiment. (B) Inhibition rate of starch digestion with or without an inhibitor. (C,D) Starch digestion kinetics were analyzed in the presence or absence of inhibitors using the multistage first‐order reaction Equation (11), represented as logarithmic slope (LOS). (E) Digestion rate constants for rapid digestion (k1) and slow digestion (k2); Blood glucose levels. (F) Schematic diagram of in vivo starch digestion experiment. (G) Fasting blood glucose level. (H) Oral glucose tolerance test. (I) Plots of HE staining results of pancreatic tissues in each group. (J) AUC of oral glucose tolerance test. (K) represents the levels of GSP in blood. (L) represents the levels of INS, GLP‐1, GIP, and DPP‐4 in blood, respectively. Differences marked with “***” indicate statistical significance at *P < 0.0001*, differences marked ****" indicate statistical significance at *P < 0.0001*.

While natural flavonoids are known to possess baseline metabolic benefits, their efficacy is often constrained by weak, reversible binding and rapid clearance. Our in vitro kinetic data (Section 2.2 and 2.3) confirmed that the SuFEx modification transforms these reversible binders into irreversible inhibitors. This shift suggests that FS6‐2 acts as a ‘pharmacological amplifier, extending target residence time in the gut. By sustaining α‐glucosidase inhibition, FS6‐2 is expected to delay carbohydrate digestion into the distal intestine more effectively than its parent compounds, thereby potentially triggering a more robust ‘ileal brake’ effect and incretin secretion (e.g., GLP‐1). To evaluate the derivative's ability to modulate postprandial glucose levels, we examined its inhibitory effects on starch digestion through comprehensive in vitro and in vivo studies (Figure 5).

The in vitro starch digestion over 180 min (Figure 5B) exhibited biphasic kinetics, characterized by a rapid digestion phase (0–30 min) followed by a slower digestion phase (30–150 min). Rate constants k1 and k2 quantified the digestion velocities for these respective phases (Figure 5C,D). Our results demonstrated that the test compounds significantly inhibited starch digestion, particularly during the initial rapid phase, as evidenced by the markedly reduced k1 and k2 values in inhibitor‐treated samples compared to controls (Figure 5E).

To assess the in vivo inhibitory effects on starch digestion, db/db mice received 28‐day treatments with FS2‐4, FS6‐2, or acarbose, with physiological parameters monitored longitudinally (Figure 5F) [29]. FS6‐2 administration produced the most pronounced reduction in both fasting and postprandial blood glucose levels (Figure 5G), demonstrating its efficacy in glycemic control. The results showed that pre‐administered inhibitors effectively attenuated the rapid blood glucose spike within 30 min after starch suspension consumption (Figure 5H). Notably, acarbose exerted a stronger initial inhibitory effect on starch digestion, whereas FS2‐4 and FS6‐2 exhibited progressively enhanced suppression over time. This divergence arises from their distinct mechanisms: Acarbose primarily inhibits α‐amylase, the enzyme that initially breaks down starch into dextrins and minimal glucose, whereas FS2‐4 and FS6‐2 selectively target α‐glucosidase, responsible for converting dextrins into absorbable glucose. The area under the curve (AUC)—reflecting cumulative glycemic response—showed significant reductions with FS6‐2 (1915.93 ± 104.40), FS2‐4 (2101.93 ± 92.26), and acarbose (2170.13 ± 111.88) vs. controls (*p* < 0.001, Figure 5J).

As the primary organ for glycemic regulation, pancreatic tissue is highly sensitive to blood glucose fluctuations. Histopathological analysis of db/db mice revealed severe pancreatic damage compared to normal controls, characterized by disrupted tissue architecture, widened interstitial spaces, prominent vacuolization, and shrunken islet cells with indistinct boundaries (Figure 5I) [30]. Treatment with acarbose, FS2‐4, or FS6‐2 significantly improved pancreatic morphology, restoring islet cell structure with clearly defined borders, reduced intercellular gaps, and elimination of vacuoles [31]. While all treatment groups demonstrated comparable restorative effects, these findings collectively suggest that glycemic control through α‐glucosidase inhibition may help preserve pancreatic integrity in diabetic conditions.

We further measured glycated serum protein (GSP) levels, which reflect average blood glucose fluctuations over 1–3 weeks. The FS6‐2 treatment group showed significantly reduced GSP levels in db/db mice compared to model. In contrast, neither FS2‐4 nor acarbose treatment produced GSP reductions relative to the model group. These results suggest that FS6‐2 may provide superior long‐term and sustained glycemic control by stabilizing blood glucose fluctuations more effectively than either FS2‐4 or acarbose (Figure 5K).

While insulin resistance (IR) remains the hallmark of type 2 diabetes mellitus (T2DM) pathogenesis, characterized by diminished tissue insulin (INS) sensitivity and impaired glucose homeostasis [32], we investigated the therapeutic potential of our inhibitors in db/db mice through comprehensive hormonal profiling. Three key findings were revealed: (I) All treatment groups (FS2‐4, FS6‐2, and acarbose) showed significant reductions in both glucose‐dependent insulinotropic polypeptide (GIP) and INS levels (Figure 5L). This metabolic improvement reduces the need for compensatory hyperinsulinemia, breaking the vicious cycle of IR progression. (II) While glucagon‐like peptide 1(GLP‐1)—which normally undergoes rapid dipeptidyl peptidase IV (DPP‐4)‐mediated degradation, was elevated across all treatments, FS6‐2 demonstrated superior efficacy [33], data (Figure 5L). This suggests that enhanced incretin activity may contribute to FS6‐2's therapeutic effects. III) Only FS6‐2 significantly increased DPP‐4 activity (Figure 5L), potentially explaining its exceptional GLP‐1 elevation through a novel regulatory mechanism [34]. These findings position FS6‐2 as a dual‐action therapeutic candidate that not only improves glucose metabolism but also significantly amplifies its potential to ameliorate diabetes‐associated metabolic dysregulation through coordinated incretin modulation.

### 2.5 SuFEx‐Modified Flavonoids Modulate Lipid Metabolism in T2DM Mice

**FIGURE 6:**
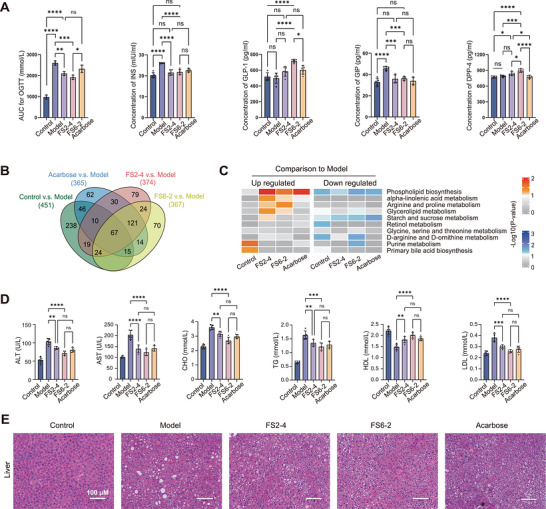
(A) Differential metabolite volcano plots of the FS2‐4, FS6‐2, and acarbose‐treated groups compared with the model group, respectively. (B) Wayne plots of the normal, FS2‐4, FS6‐2, and acarbose‐treated groups compared with the model group, respectively. (C) Metabolite pathway clustering analysis of the normal, FS2‐4, FS6‐2, and acarbose‐treated groups compared with the model group, respectively. (D) Corresponding to blood levels of TG, CHO, HDL, and LDL, respectively. (E) Corresponding to blood levels of ALT and AST, respectively. (F) The HE staining results of liver tissues in each group.

We further conducted widely targeted metabolomics (UPLC‐MS) comparing normal, FS2‐4, FS6‐2, and acarbose groups to the model group to elucidate treatment‐specific metabolite patterns. All three compounds significantly altered the serum metabolite profile. Martynoside levels increased significantly in response to all three compounds, whereas choline and leukotriene C5 levels increased only in response to FS2‐4 and acarbose (Figure 6A). Differential analysis revealed 451 (Control/Model), 374 (FS2‐4/Model), 367 (FS6‐2/Model), and 365 (Acarbose/Model) metabolites, with predominant alterations in lipid and starch metabolism pathways (Figure 6B,C). Collectively, these findings suggest that FS4‐2 and FS6‐2 show potential for regulating lipid metabolism among the compounds tested.

Disorders of glucose and lipid metabolism are pathophysiologically linked. Insulin resistance impairs cellular glucose uptake, resulting in hyperglycemia, while elevated circulating free fatty acids derived from adipose tissue further impair insulin signaling. Conversely, glycerol liberated during lipolysis serves as a gluconeogenic precursor. This reciprocal dysregulation establishes a vicious cycle, necessitating integrated therapeutic strategies [31, 35, 36]. To demonstrate the function of FS6‐2 in lipid metabolism, we assessed key lipid metabolism parameters in db/db mice. All treatment groups exhibited significant reductions in blood lipid levels compared to untreated controls, including lowered triglycerides (TG), total cholesterol (CHO), and small dense low‐density lipoproteins (LDL), alongside increased high‐density lipoprotein (HDL) (Figure 6D).

Given the liver's central role in lipid synthesis, storage, and redistribution [37], we examined liver histology. The analysis revealed that diabetes‐induced lipid disorders resulted in hepatocyte swelling and the formation of fat vacuoles, whereas all treatment groups showed restored liver architecture and reduced lipid accumulation (Figure 6F). FS6‐2 exhibited greater efficacy than acarbose in reducing fat vacuoles. These improvements in dyslipidemia markers were paralleled by a decrease in hepatic injury, as evidenced by significantly reduced aspartate aminotransferase (AST), and alanine aminotransferase (ALT) levels (Figure 6E). Emerging evidence suggests that oxidative stress modulates the expression and activity of enzymes involved in lipid metabolism, thereby disrupting cellular lipid homeostasis [38]. To investigate the molecular basis of oxidative stress, we assessed antioxidant levels in serum and metabolism‐related organs such as the liver and muscle. Antioxidant indices were pointedly elevated in all treatment groups compared to untreated diabetic mice (Figure S8) [35, 39, 40]. In contrast to the liver, no obvious alternation was observed in the other organs (Figure S9).

### 2.6 SuFEx‐Modified Flavonoids Remodel Gut Microbiota in T2DM Mouse

**FIGURE 7:**
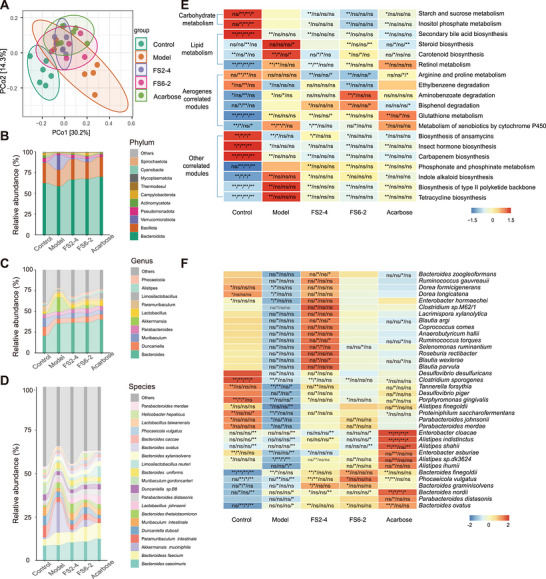
Macrogenomics analysis. (A) Plot of PCA analysis for each treatment group. (B) Phylum‐level species richness. (C) Genus‐level species richness. (D) Species richness at the species level. (E) Analysis of microbial metabolite pathway enrichment caused by modeling and intervention (F) Model‐induced differentially expressed strains associated with gas production and their recovery after intervention.

The nonselective therapeutic agents (e.g. acarbose) often result in off‐target effects, including perturbations to fecal microbiota homeostasis, which may manifest clinically as gastrointestinal disturbances (e.g., bloating and diarrhea) [37, 41, 42]. To evaluate the microbiota‐modulating potential of lead inhibitors, we conducted a comparative analysis of gut microbial communities in normal and diabetic db/db mice (Figure S10). α‐Diversity metrics (Shannon, Simpson, Chao1, and Observed Species indices) revealed a significant reduction in microbial richness and diversity in db/db mice relative to controls (*p < 0.05*), while microbial richness recovered due to the treatment (Figure S11). Figure S10E illustrates that the abundance of 34 bacterial strains was reduced in the model mouse in comparison with controls and recovered in the treatment groups. Another 8 bacterial strains were increased in the model mouse and further altered in the treatment groups. β‐Diversity analysis (PCoA, weighted UniFrac) further demonstrated distinct compositional clustering of colonic microbiota among the five experimental groups (Figure 7A). At the phylum level, the Model group displayed a pronounced decline in Bacteroidota and Bacteroides abundance (p < 0.01) (Figure 7B) [43]. Genus‐level and species‐level taxonomic profiling further highlighted differential microbial community structures across groups, underscoring the specificity of intervention‐related microbiota modulation (Figure 7C,D). Notably, *Akkermansia* abundance was elevated in the gut microbiota of diabetic mice compared with normal controls but was significantly reduced following treatment with α‐glucosidase inhibitors.

Functional analysis of the gut microbiome revealed that carbohydrate and lipid metabolism were significantly altered in the treatment groups after intervention (Figure 7E). Compared to Acarbose treatment, FS2‐4 and FS6‐2 markedly downregulated modules associated with the gas‐producing process (e.g., amino acid metabolisms) (Figure 7E). Focusing specifically on gas‐producing microbiota, a striking difference emerged between the effects of Acarbose and the flavonoid‐derived compounds (Figure 7F). Acarbose significantly increased the abundance of *Alistipes*, *Enterobacter*, *Bacteroides*, *Phocaeicola*, and *Parabacteroides*, FS2‐4 upregulated the abundance of *Dorea*, *Blautia*, *Ruminococcus*, etc., respectively. Notably, FS6‐2 had a moderate influence on the alternation of gas‐producing fecal microbiota.

**FIGURE 8:**
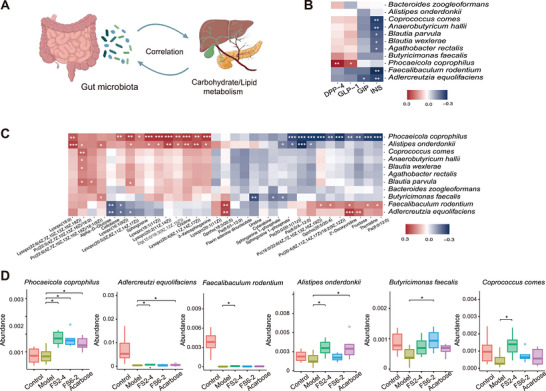
Analysis of differential bacterial colony expression and correlation with metabolites. (A)Diagram of the relationship between gut microbiota and carbohydrate /lipid metabolism. (B) Correlation analysis of differential bacterial strains with intestinal hormones. (C) Correlation analysis of differential strains with metabolites of lipid metabolism and carbohydrate metabolism pathways. (D) represent the abundance of *Phocaeicola_coprophilus, Adlercreutzia_equolifaciens, Faecalibaculum_rodentium, Alistipes_onderdonkii, Butyricimonas virosa, and Coprococcus comes*.

The variation in gut microbiota significantly affected the serum concentrations of INS, DPP‐4, GLP‐1, and GIP, which are indices related to carbohydrate metabolism, highlighting a microbiota–endocrine axis in the regulation of carbohydrate metabolism (Figure 8A,B) [41]. Some species, such as *Phocaeicola coprophilous*, *Alistipes onderdonkii*, *Adlercreutzia equolifaciens*, etc., strongly influence the serum lipid metabolism, consistent with their reported roles in bile acid metabolism, lipid absorption, and hepatic lipid homeostasis (Figure 8C,D). These findings suggest that SuFEx‐modified flavonoids significantly enhance and prolong systemic metabolic benefits not only via direct enzyme inhibition but also through microbiota‐mediated modulation of host endocrine and lipid metabolic pathways. It is noteworthy that natural flavonoids intrinsically possess baseline glucose‐ and lipid‐regulating activities [44]. In this context, SuFEx‐derived ‐OSO_2_F modification appears to function as a pharmacological enhancer. By forming a stable covalent bond with α‐glucosidase, FS6‐2 may prolong target engagement beyond the rapid dissociation typically observed for the parent flavonoids. Such sustained inhibition could delay carbohydrate digestion and absorption, potentially extending these processes toward the distal intestine and thereby contributing to activation of the ileal brake and secretion of incretins such as GLP‐1 [41, 45, 46]. Accordingly, the systemic metabolic benefits observed, including improved lipid profiles and gut microbiota modulation, may reflect the combined contribution of the intrinsic flavonoid scaffold and the amplified physiological effects associated with prolonged covalent inhibition [4].

## 3 Discussion

The ability of covalent targeting to lower effective doses and prolong therapeutic action has established it as a key strategy in modern drug discovery [7]. This has prompted the exploration of diverse covalent warheads to improve inhibitor potency and selectivity. In our study, the SuFEx‐modified flavonoids FS6‐2 and FS2‐4 exhibited markedly improved affinity toward α‐glucosidase compared with their unmodified parent substrates. Because α‐glucosidase operates in the gastrointestinal tract—an environment characterized by fluctuating pH from the oral cavity to the intestine—a covalent warhead with controlled reactivity is particularly advantageous. SuFEx chemistry was therefore selected for inhibitor design based on several key merits. First, compared with classical warheads such as acrylamides or epoxides, SuFEx electrophiles display moderate and tunable reactivity, enabling selective reaction with nucleophilic residues (e.g., Lys, Thr) while minimizing nonspecific protein modification [9, 47]. More importantly, among the diverse range of SuFEx‐enabled linkers, aryl fluorosulfates (‐OSO_2_F) offer an optimal balance between aqueous stability and proximity‐driven reactivity [16]. As highlighted in recent reviews, although sulfonyl fluorides (─SO_2_F) are highly electrophilic and widely used, they may be more prone to premature hydrolysis or nonspecific off‐target labeling in complex biological environments. Likewise, primary sulfamoyl fluorides (‐NHSO_2_F) can exhibit limited stability under basic conditions [17, 48]. In contrast, the ‐OSO_2_F group remains exceptionally stable and largely inert within the fluctuating pH environment of the gastrointestinal tract. This controlled reactivity is critical for suppressing off‐target effects that often arise from overly reactive covalent modifiers. Moreover, by limiting unintended protein alkylation, SuFEx‐based covalent engagement is expected to reduce biological toxic side effects, an important consideration for chronic metabolic intervention. Consistent with this expectation, long‐term treatment in mice showed no apparent adverse effects on hematological parameters or major organs (Figure S9). This balance improves target selectivity and reduces off‐target risks. Second, SuFEx reactions proceed efficiently in aqueous and diverse physiological conditions, making them well‐suited for the dynamic gastrointestinal environment and for orally delivered or gut‐localized therapeutics [47, 49, 50]. Third, SuFEx linkages are stable and hydrolysis‐resistant, enabling durable target engagement and sustained in vivo efficacy [49]. For oral covalent inhibitors, however, the stability and safety of the unreacted warhead are equally critical. In contrast to conventional electrophiles that are often highly reactive and hydrolysis‐prone[7], aryl fluorosulfates (‐OSO_2_F) display exceptional kinetic stability across aqueous and physiological pH conditions. Their S(VI)─F bond resists nonspecific attack and is activated mainly through proximity‐driven interactions within a target protein active site [16]. This feature minimizes spontaneous hydrolysis and unintended release of toxic species in the gastrointestinal tract. Moreover, SuFEx warheads are generally less cytotoxic than classical covalent groups [7]. Consistently, our 28‐day in vivo study showed no detectable adverse effects on body weight, organ indices, or tissue histology (Figure S9). These results highlight the stability and safety of the ‐OSO_2_F moiety and support its suitability for oral precision metabolic therapeutics.

Having established the advantages of SuFEx chemistry for gut‐localized covalent inhibition, we next require understanding the molecular basis for the pronounced selectivity toward α‐glucosidase over α‐amylase. The selective inhibition of α‐glucosidase by SuFEx‐modified flavonoids, as opposed to α‐amylase, can be attributed to two key factors: active‐site chemistry and pocket topology. First, α‐glucosidase contains a well‐positioned nucleophile, particularly the accessible lysine within the pocket, which facilitates the electrophilic attack of the fluorosulfate group on SuFEx‐modified flavonoids [47]. This interaction forms a stable covalent bond between the inhibitor and the enzyme, leading to potent and sustained inhibition. In contrast, α‐amylase lacks a similarly positioned reactive residue (e.g., Lys), making it less subject to SuFEx modification [51]. Additionally, the distinct pocket topologies of the two enzymes further explain their differential inhibition. α‐glucosidase has a relatively small, hydrophobic binding pocket that favors small aromatic ligands like flavonoids [52, 53]. This enables the SuFEx‐modified flavonoids to fit snugly into the active site, enhancing selectivity and binding affinity. On the other hand, α‐amylase features a larger, more open binding site designed to accommodate bulky polysaccharides, resulting in poor shape complementarity that prevents the formation of stable intermolecular interactions with smaller, aromatic ligands [51, 54]. Thus, both the favorable nucleophilic environment in α‐glucosidase and the enzyme's compact binding pocket contribute to the improved specificity of SuFEx‐modified flavonoids as selective α‐glucosidase inhibitors.

Given that the biochemical assays were conducted using yeast‐derived α‐glucosidase, it was critical to evaluate the translational relevance of these mechanistic findings. The biochemical assays in this study utilized *Saccharomyces cerevisiae* α‐glucosidase, a long‐standing discovery platform that offers a structurally conserved catalytic core and allows for the precise interrogation of warhead–nucleophile reactivity. Although yeast and mammalian α‐glucosidases differ in certain regulatory and substrate‐recognition features, the preservation of the catalytic Asp/Glu nucleophile renders the yeast model particularly informative for assessing covalent chemistries such as SuFEx [55]. Recognizing the intrinsic cross‐species differences, we strategically integrated in vivo efficacy studies and gut‐microbiota multi‐omics profiling to establish physiological relevance. The robust improvements in glycemic control, together with microbiota remodeling consistent with suppressed carbohydrate digestion, demonstrate that the covalent inhibition captured in the yeast system manifests coherently at the organismal level. This integrative validation framework underscores that the yeast enzyme serves as a mechanistically robust and translationally meaningful platform for early discovery of covalent α‐glucosidase inhibitors.

Beyond direct enzyme inhibition, selective suppression of intestinal α‐glucosidase is expected to reshape carbohydrate metabolism, with downstream consequences for gut microbiota. The SuFEx‐modified compounds have also modulated specific microbial populations. Remarkably, Akkermansia abundance was elevated in the gut microbiota of diabetic mice compared with normal controls but was significantly reduced following treatment with α‐glucosidase inhibitors (Figure 7). *Akkermansia muciniphila* preferentially proliferates in mucin‐rich environments, which are frequently associated with increased intestinal permeability and compromised barrier integrity under diabetic conditions [56]. Upon pharmacological control of postprandial hyperglycemia, intestinal metabolic homeostasis was progressively restored, accompanied by a reconfiguration of the gut microbial community. The reduction in *Akkermansia* abundance after treatment likely reflects an improvement in gut barrier function and normalization of mucin dynamics, rather than a direct inhibitory effect, underscoring the close interplay between glycemic regulation and microbiota composition. Furthermore, FS2‐4 and FS6‐2 selectively modulated specific microbial populations, leading to a shift toward beneficial bacteria such as *Dorea*, *Blautia* and *Ruminococcus*, while simultaneously reducing the abundance of gas‐producing species like *Alistipes* and *Bacteroides*. This selective microbial modulation contrasts with the effects of acarbose, which promoted an overgrowth of gas‐producing bacteria, potentially leading to gastrointestinal side effects. These findings suggest that SuFEx‐modified flavonoids offer a more targeted approach to microbiota regulation without the adverse consequences commonly associated with non‐selective inhibitors. These microbiota‐driven changes correlate with significant metabolic improvements, including enhanced glycemic control and lipid metabolism (Figure 8). The ability of FS2‐4 and FS6‐2 to restore microbial balance and regulate specific microbial populations highlights the broader therapeutic potential of SuFEx modification in managing diabetes and metabolic diseases through integrated microbiota modulation.

We acknowledge that, although the unmodified parent compounds (F2 and F6) were included in our earlier in vitro and preliminary in vivo evaluations and showed weaker activity than the corresponding SuFEx‐modified derivatives, they were not carried forward into the subsequent 28‐day efficacy study. That later animal experiment was specifically designed to benchmark the optimized lead FS6‐2 against the clinical standard acarbose and to assess its translational potential as a precision therapeutic. Therefore, while our current dataset supports the superiority of SuFEx modification at the screening and initial validation stages, it does not yet provide a direct long‐term in vivo comparison between FS6‐2 and its parent scaffold under the same treatment framework. Future comparative pharmacodynamic studies will be needed to more precisely distinguish the intrinsic contribution of the flavonoid scaffold from the additional in vivo benefits conferred by the covalent SuFEx warhead. In summary, this work demonstrates that SuFEx‐enabled covalent reprogramming effectively converts natural flavonoids into highly selective α‐glucosidase inhibitors with systemic metabolic benefits. Incorporation of fluorosulfate warheads enables precise covalent engagement of the enzyme active site, markedly enhancing inhibitory potency and selectivity. Beyond enzyme inhibition, SuFEx‐modified flavonoids also improve glycemic and lipid homeostasis in diabetic mice and restore gut microbial diversity, accompanied by coordinated modulation of incretin signaling and insulin dynamics. These findings integrate click chemistry, covalent enzyme targeting, and microbiota‐mediated metabolic regulation, establishing a generalizable strategy for transforming dietary natural products into selective metabolic therapeutics [57, 58].

One limitation of the present study is the relatively limited throughput of compound evaluation. In this work, 11 representative flavonoids were selected to demonstrate the feasibility of designing covalent α‐glucosidase inhibitors through SuFEx‐enabled flavonoid reprogramming. Although this proof‐of‐concept strategy successfully identified active covalent candidates, the chemical space explored remains limited, particularly considering that more than 8000 naturally occurring flavonoids with diverse structural features have been documented. In future studies, the throughput of this platform could be substantially increased by incorporating high‐throughput screening technologies, such as DNA‐encoded libraries, or AI‐guided molecular design models. These approaches would enable the rapid generation, prioritization, and validation of larger covalent flavonoid libraries with improved potency, selectivity, and drug‐like properties.

## 4 Methods

### 4.1 Materials

α‐glucosidase (EC 232‐604‐7, lyophilized powder, ≥100 units/mg protein) isolated from *Saccharomyces cerevisiae* and α‐amylase (EC 232‐565‐6, powder, ≥5 units/mg solid) isolated from porcine pancreas were purchased from Sigma–Aldrich Co. (St. Louis, USA). All reagents were commercially available and used without further purification. Parent flavonoid compounds (F1–F11), including F2 and F6, were purchased from Macklin Biochemical Co., Ltd. (Shanghai, China) with an analytical purity of ≥98% as determined by the manufacturer.

### 4.2 General Instrumentation and Analytical Methods

^1^H and ^19^F nuclear magnetic resonance (NMR) spectra were recorded on an Agilent‐400 instrument at 400 MHz and 376 MHz, respectively. ^13^C NMR spectra were recorded on a Bruker AM‐400 instrument at 101 MHz. Chemical shifts (δ) were expressed in parts per million (ppm) relative to residual solvent as an internal reference for ^1^H and ^13^C NMR. Coupling constants (J) were reported in hertz units (Hz) and coupling patterns were described as singlet (s), doublet (d), triplet (t), quartet (q), multiplet (m), and broad (br). High resolution mass spectra (HRMS) were carried out on an Agilent Technologies 6230 LC‐MS with (ESI‐TOF) mode, a Thermo Fisher Scientific LTQ FTICR‐MS instrument with direct analysis in real time (DART) mode, or on a Waters Micromass GCT Premier with electron impact (EI) ionization mode. The final purity of the synthesized key compounds was determined to be ≥90% using a High‐Performance Liquid Chromatography (HPLC) system (Agilent 1260 Infinity) equipped with a reversed‐phase C18 column, ensuring their suitability for subsequent biological evaluations.

### 4.3 General Procedures for Preparing Target Compounds

Synthesis of target compounds was shown in flowchart 1 [17]. In simple terms, compound a–k (1 equiv), and fluorosulfuryl imidazole salt K (1.3 equiv) are added to a 100 uL MeCN solution, and then Et_3_N (1.6 equiv) is added to catalyze the reaction. Alternatively, the reaction ratio was adjusted so that compound a–k (1 equiv) and fluorosulfuryl imidazole salt K (2.6 equiv) are added to a 100 uL MeCN solution, and then Et_3_N (3.2 equiv) is added to catalyze the reaction. The mixture was reacted at room temperature for 1 h. After the reaction was completed, the reaction mixture was dried and separated by silica gel column chromatography using petroleum ether/ethyl acetate (5:1, v/v) as the developing agent.

### 4.4 Methods for Library Construction

In a 96‐well plate, the compound library (0.01 mmol) was added to MeCN (100 µL) of fluorosulfuryl imidazole salt, and triethylamine was added for catalytic reaction, and the reaction was shaken at room temperature overnight. Adjust the substitution ratio of ‐OSO_2_F according to the number of hydroxyl groups in the compound, and set the ratio of compound, sulfonate, and triethylamine to (1:1.3:1.6), (1:2.6:3.2), (1:3.9:4.8), (1:5.2:6.4), (1:6.5:8.0), or (1:7.8:9.6). The library solution was diluted with DMSO to 5mm and continuously diluted twice to measure inhibition. A single solvent hole (without a natural product library) was set up in the 96‐well plate as a blank control, and the results showed that the enzyme activity was not affected under the solvent condition (Figure S1).

### 4.5 In Vitro Enzyme Inhibition Assay

In vitro α‐glucosidase inhibition assay: α‐glucosidase inhibitory activity was detected using the method of Zeng, L et al. with slight modifications [59]. Dilution solution (10 µL), α‐glucosidase solution (0.25 U/mL, 40 µL), and PBS (0.1 m, Ph6.8, 100 µL) were mixed on a 96‐well plate and incubated for 5min at 37°C. Then 50 µL pNαGP (0.6 mm) was added, and the reaction was placed at 37 °C for 10 min. The absorbance value of the reaction liquid was measured at 405 nm to reflect the activity of the reaction liquid. Acarbose was used as a positive control, and other solvent mixtures without reactants were used as a blank control.

In vitro α‐amylase inhibition assay: α‐amylase inhibitory activity was detected using the method of Zeng, L et al. with slight modifications [59]. Dilution solution (10 µL), α‐amylase solution (5 U/mL, 40 µL), and PBS (0.1 m, Ph 6.8, 100 µL) were mixed on a 96 well plate and incubated for 5 min at 37°C. Then 50 µL G3‐CNP (0.5mm) was added, and the reaction was placed at 37°C for 10 min. The absorbance value of the reaction liquid was measured at 405 nm to reflect the activity of the reaction liquid. Acarbose was used as a positive control, and other solvent mixtures without reactants were used as a blank control.

The inhibition effect was calculated by the following equation:

(1) $$\begin{array}{ccc} & & \alpha -\mathrm{glucosidase}\;\mathrm{inhibition}\;\mathrm{rate}\;\left(\%\right)\\ & & \;=\frac{\mathrm{Abs}\;\mathrm{control}-\mathrm{Abs}\;\mathrm{sample}}{\mathrm{Abs}\;\mathrm{control}}\times 100 \end{array}$$

The IC_50_ values of the tested compounds were calculated using nonlinear fitting (logit method).

### 4.6 Inhibition Kinetic Studies

The inhibition kinetics were determined by the same method as the in vitro α‐glucosidase inhibition assay, with only the concentration of substrate pNαGP changed to 0.9, 1.2, 1.5, and 3.0mm [59]. To determine the type of inhibition, the Michaelis‐Menten constant (*K_m_*) was calculated by plotting Lineweaver‐Burk plots of substrate concentration (1/[*S*]) and enzyme velocity (1/*V*) at different inhibitor concentrations (Equation 2). The constant value (*K_i_*) of the experimental inhibitor was obtained by the conic curve of inhibitor concentration [*I*] and Km (Equation 3).

(2) $$\frac{1}{V}=\frac{K_{m}}{V_{max}\left[I\right]}+\frac{1}{V_{max}}$$

(3) $$Slope=\frac{K_{m}}{V_{max}}+\frac{K_{m}}{V_{max}\cdot K_{i}}\left[I\right]$$

where *v* is the initial reaction velocity, *V_max_* is the maximum initial reaction velocity, [*I*] is the concentration of the inhibitor, and *K_m_* is the Michaelis constant.

The Dixon equation was applied to calculate *K_ic_* [60]. The equation for competitive inhibition is written as follows:

(4) $$v=\frac{V_{max}\cdot a}{K_{m}\left(1+\frac{\left[I\right]}{K_{ic}}\right)+a}$$

The equation for mixed inhibition is written as follows:

(5) $$v=\frac{V_{max}\cdot a}{K_{m}\left(1+\frac{\left[I\right]}{K_{ic}}\right)+a\left(1+\frac{\left[I\right]}{K_{iu}}\right)}$$

where *a* is the concentration of the starch, *K_ic_* is the competitive inhibition constant, and Kiu is the uncompetitive inhibition constant. *K_ic_* is equal to the absolute value of the abscissa of the intersection between Dixon plots at different substrate concentrations.

Although Dixon plots cannot directly give the value of *K_iu_*, a similar derivation reveals that this value can be found by plotting a/v against [*I*] at several a values to produce an Eisenthal‐Cornish‐Bowden plot [61]. The full Cornish‐Bowden equation for mixed inhibition can be expressed as follows:

(6) $$v/a=\frac{V_{max}}{K_{m}\left(1+\frac{\left[I\right]}{K_{ic}}\right)+a\left(1+\frac{\left[I\right]}{K_{iu}}\right)}$$

*K_iu_* equals the absolute value of the abscissa of the intersection between Cornish‐Bowden plots at different substrate concentrations.

### 4.7 Fluorescence Quenching Experiment

The fluorescence quenching experiment was modified to some extent based on previous methods [62, 63]. α‐glucosidase (1.0 mL, 2 U/mL) was titrated with different concentrations of inhibitor solution (1.0 mL and 0–500 mm), and fluorescence determination was performed after standing equilibrium at 300.15, 305.15, or 315.15 K for 5 min, respectively. The emission wavelength (λ) of the fluorescence spectrum is 300–500 nm, the excitation wavelength (λ) is 280 nm, and the peak width is 5 nm. Fluorescence quenching analysis based on the Stern‐Volmer Equation (7).

(7) $$\frac{F0}{F}=1+K_{q}\tau 0\left[Q\right]=1+K_{sv}\left[Q\right]$$

F and F0 are the fluorescence intensity with and without inhibitors, *K_sv_* is the Stern–Volmer quenching constant, *K_q_* is the bimolecular quenching constant, and [*Q*] is the concentration of inhibitors.

When the quenching type conforms to static quenching, the binding constant *K_a_* and the number of binding sites for each enzyme can be calculated by Equation (8).

(8) $$log\frac{F0-F}{F}=logK_{a}+nlog\left[Q\right]$$

*K_a_* is the binding constant and n is the number of binding sites.

Furthermore, the thermodynamic parameters and the type of forces between enzyme and inhibitors are analyzed by Vant't Hoff Equation (9).

(9) $$\log K_{a}=-\frac{\Delta H}{2.303RT}+\frac{\Delta S}{2.303R}$$

(10) ΔG=ΔH−TΔS

*K_a_* is the binding constant, R is the universal gas constant, and T represents the test temperature (300.15, 305.15, 310.15K). The thermodynamic parameters ∆H, ∆S, and ∆G represent the change in heat, entropy, and free energy, respectively.

### 4.8 Synchronous Fluorescence Spectra

Synchronous fluorescence spectra can be used to reflect the changes in conformation and microenvironment near protein fluorophores. When Δλ is set to 15 and 60 nm, it can represent changes in tyrosine and tryptophan residues, respectively [23, 64]. α‐glucosidase (1.0 mL, 2 U/ mL) was titrated with different concentrations (1.0 mL, 0–500 mm) of inhibitor solution, and fluorescence determination was performed after equilibrium for 5 min. Synchronous fluorescence spectra at different temperatures (305.15, 310.15, and 315.15 K) were measured with the F‐7000 fluorescence spectrophotometer. The wavelength spacing is Δλ = 15 nm and Δλ = 60 nm, the excitation wavelength range is 260–400 nm, and the excitation and emission slit widths are set to 5.0 nm. The synchronous fluorescence quenching ratio (RSFQ) was calculated using the following Equation (11):

(11) $$RSQF=1-F/F_{0}$$

### 4.9 Three‐Dimensional Fluorescence Spectra

The compound (1.0mL and100 mm) was added to α‐glucosidase (1.0mL and 2U/ mL) and incubated for 5 min. The 3D fluorescence spectra of the mixture were recorded by the F‐7000 fluorescence spectrophotometer. The excitation wavelength (200‐350nm), the emission wavelength (250–400 nm), excitation interval (10 nm), emission interval (10 nm), and the slit width (5 nm) were set, respectively. Import the data into the origin for processing [23].

### 4.10 Surface Plasmon Resonance (SPR) Biosensor Assays

The SPR experiments were conducted at room temperature (25°C) using the Biacore 8 k^+^ instrument and sensor chip CM5 (Cytiva, catalog No. 29149603) [65, 66]. Initially, the NHS and EDC activating groups were injected onto the chip surface to activate it. Subsequently, α‐glucosidase was coupled to the activated chip surface through the amino coupling method, followed by sealing of the chip with ethanolamine. After fixation, compounds with concentrations ranging from 500 to 0.390625 µm were injected onto the chip surface at a flow rate of 30 µL/min in PBST (1 mm PBS, 0.05% Tween‐20 PH 6.8) containing either 5% DMSO or Tween‐Tris‐HCl (1 mm Tris‐HCl, 0.05% Tween‐20 PH 8.0) containing either 5% DMSO. The combined phase monitoring lasted for 90 s while separated phase monitoring lasted for100 s. Four‐point solvent correction was performed using different concentrations of DMSO solutions (5.8%, 5.3%, 4.9%, and 4.5%). The data obtained were analyzed using Biacore insight evaluation software (Cytiva, Uppsala, Sweden). The sensor image was corrected by subtracting a blank injection signal. To determine the KD value, the steady‐state signal point at the end of injection was fitted using nonlinear regression analysis based on either a reversible one‐step 1:1 binding interaction model equation or a two state reaction equation.

### 4.11 Time‐Dependent Inhibition Assay

To evaluate the time‐dependence of FS6‐2 inhibition, the α‐glucosidase (0.1 U/mL) was pre‐incubated with various concentrations of FS6‐2 (0, 4, 8, 16, 32, 64, and 128 µm) in 0.1 m PBS (pH 6.8) at 37°C for different durations (0, 15, 30, 45, and 60 min). Following pre‐incubation, the enzymatic reaction was initiated by adding 50 µL of pN αPG substrate (final concentration maintained at 0.6 mm to ensure consistency with previous datasets). The reaction was terminated after 15 min by adding 50 µL of 0.2 m Na_2_CO_3_, and the absorbance was measured at 405 nm. The IC_50_ values for each time point were calculated to observe the inhibitory potency shift [26].

### 4.12 Reversibility Assay (Jump‐Dilution)

The reversibility of the enzyme‐inhibitor complex was determined using a 100‐fold jump‐dilution method. A high‐concentration enzyme stock (10 U/mL, 100× working concentration) was pre‐incubated with FS6‐2 (160 µm, 10 × IC_50_) or Acarbose (5.6 mm, 10 × IC_50_) at 37°C for 60 min to ensure saturated binding. The mixture was then rapidly diluted 100‐fold into the assay buffer containing pNPG (final substrate concentration 0.6 mm). The residual enzyme activity was measured at specified time intervals (0, 15, 30, 60, and 120 min) following dilution. The recovery of enzyme activity was monitored and compared to a vehicle control (DMSO) to distinguish between reversible and irreversible inhibition [26].

### 4.13 Identification of Covalent Modification Sites by MS/MS Peptide Mapping

To pinpoint the specific residue modified by FS6‐2, the α‐glucosidase protein (1 mg/mL) was incubated with FS6‐2 (100 µm) or vehicle (DMSO) in 0.1 m PBS (pH 6.8) at 37°C for 2 h to ensure complete covalent engagement. After incubation, the protein was denatured in 8 m urea and reduced with 10 mm dithiothreitol at 56°C for 45 min, followed by alkylation with 50 mm iodoacetamide in the dark for 30 min. The samples were then diluted to < 1 m urea using 50 mm ammonium bicarbonate and digested with sequencing‐grade trypsin (Promega) at a 1:50 (w/w) ratio at 37°C overnight.

The resulting peptide mixtures were desalted using C18 ZipTips and analyzed by nano LC‐MS analysis using the nano‐Acquity nano HPLC (Waters, Milford, MA, USA) coupled with a Thermo Q‐Exactive high‐resolution mass spectrometer (Thermo Scientific, Waltham, MA, USA). The covalent modification was defined as a dynamic mass shift corresponding to the FS6‐2 molecular weight minus the leaving HF group on nucleophilic residues, including Lys, Tyr, Ser, and Thr [67]. A false discovery rate (FDR) of < 1% was applied for peptide identification.

### 4.14 Construction and Expression of Yeast‐Derived α‐Glucosidase Mutant and SPR Biosensor Assays

A yeast‐derived α‐glucosidase gene was synthesized and cloned into the pET‐28 vector via homologous recombination using BamHI and XhoI restriction sites. To introduce the K480G mutation, two pairs of primers with overlapping sequences at the residue 480 site were designed. The target mutation (Lys to Gly) was achieved through PCR amplification followed by seamless assembly to generate the mutated pET‐28 plasmid.

The fragment encoding the mutant protein was subsequently subcloned into the Addgene 169887 vector. The resulting construct was transformed into competent E. coli cells. Single colonies were inoculated into TB medium and cultured overnight at 37°C, followed by cooling to 18°C for induction. Protein expression was triggered by the addition of IPTG. After harvesting the biomass via centrifugation, the recombinant protein was purified using a Ni‐NTA affinity column and eluted with an imidazole gradient. This workflow effectively combined site‐directed mutagenesis with a low‐temperature induction strategy to yield the purified α‐glucosidase mutant. The mutated α‐glucosidase was fixed using the same method, and the KD values of FS6‐2 and the mutated α‐glucosidase were measured.

### 4.15 Broad Digestive Enzyme Selectivity Profiling

To rigorously evaluate the potential off‐target covalent reactivity of the SuFEx warhead in the gastrointestinal tract, the inhibitory activity of FS6‐2 was tested against a panel of major digestive enzymes, including pepsin (an acidic gastric protease), trypsin (an intestinal serine protease), and pancreatic lipase. All enzymes were pre‐incubated with a high challenge concentration (200 µm) of FS6‐2, Acarbose, or their respective specific positive controls at 37°C for 15 min. The positive controls included Pepstatin A (1 µm) for pepsin, freshly prepared PMSF (1 mm) for trypsin, and Orlistat (50 µm) for pancreatic lipase [7]. Following pre‐incubation, the corresponding substrates were added to initiate the reactions according to standard enzymatic assay protocols. The relative remaining enzyme activity of the FS6‐2 and Acarbose groups was calculated by comparing them to the vehicle control (DMSO) to determine target selectivity.

### 4.16 Molecular Docking Simulation

In this experiment, AutoDock Vina software was used for molecular docking [68]. The first step is to prepare the protein crystal structure 2QMJ was selected from the protein database as the crystal structure of α‐glucosidase, and the protein crystal structure was subsequently subjected to water molecular removal and hydrotreatment. The second step is the preparation of small molecules. Use ChemBiodraw Ultra 14.0 to map the inhibitor's three‐dimensional structure, minimizing the energy of small molecules. The third step is to generate a docking box for the center and use AutoDock Vina for docking. Ten poses were generated during the docking process, and the pose with the highest Glide score was selected for the subsequent study on the interaction between inhibitors and α‐glucosidase.

### 4.17 Molecular Dynamics Study

Molecular dynamics (MD) simulations using AmberTools 20 packets on the Yinfo cloud computing platform (https://cloud.yinfotek.com/). The specific methods were adopted as previously reported by the research group [69].

### 4.18 In Vitro Test in Starch Model

The effect of inhibitors on starch digestibility in vitro was determined by the Englyst method with slight modifications [70, 71]. Cornstarch (300 mg) and guar gum (25 mg) were added into a 50 mL centrifuge tube and dissolved with 7.5mL distilled water. It was then boiled in a boiling water bath for 10 min, cooled to room temperature, and then sodium acetate buffer (2.5 mL, 0.4 m, pH 5.2, and containing 0.18% (w/v) CaCl_2_). The sample tube was equiliblated at 37°C for 15 min before adding fresh porcine trypsin extract, starch glucosidase, and inhibitor mixture (5.5 mL) to hydrolyze starch. At the same time, the groups without inhibitor and acarbose were set as blank and positive control. At 20, 60, 120, and 240 min, 250 µL starch hydrolysate was taken from the centrifuge tube and added to 10.0 mL 66% (v/v) ethanol. The glucose generation was measured with the D‐glucose assay kit (GOPOD).

### 4.19 In Vivo Inhibition of Starch Digestion

After in vitro validation, to further characterize the inhibitor's effect on postprandial blood glucose control, in vivo characterization was conducted through intragastric administration in mice [72]. The db/db mice and C57/BL mice aged 5–6 weeks were utilized as control groups in the experiment. Prior to the formal experiment, they were acclimated to the experimental environment for 1 week [73]. The animal experiments were conducted in a specific pathogen‐free (SPF) environment. The Experimental Animal Management and Welfare Ethics Committee of Beijing Huayuan Times Technology Co., LTD. approved the study (Ethics Review batch number: HYSD2023‐04). The db/db mice were randomly divided into four groups: diabetes model control group, acarbose group, FS2‐4 group, and FS6‐2 group, with eight mice in each group. FS2‐4, FS6‐2, and acarbose were dissolved in normal saline containing 10% DMSO. Group FS2‐4 received intragastric administration at a dose of 20 mg/kg while labeled as such. Group FS6‐2 was administered intragastrically at a dose of 20 mg/kg and labeled accordingly. The acarbose group received intragastric administration at a dose of 20mg/kg and was labeled as the acarbose group. Mice in the normal control group and diabetic model group received an equivalent amount of normal saline via intragastric administration or oral gavage respectively; these groups were labeled Control group and Model group respectively. Throughout the continuous 4‐week period of intragastric administration, all mice had ad libitum access to food and water intake was recorded weekly along with their weight measurements and fasting blood glucose levels.

After administration, the mice were anesthetized for orbital blood collection. The collected blood was then centrifuged at a low temperature of 3500 r/min and after 10 min, the serum was isolated and divided into frozen storage tubes. Following blood collection, the mice were euthanized by neck dislocation and dissected to obtain heart, liver, lungs, kidneys, pancreas, spleen, and muscle tissue samples. Any blood stains on the tissues were washed with normal saline solution before excess water on their surfaces was removed using filter paper. Organ indices were calculated accordingly. A portion of each pancreas, kidney, liver, and spleen tissue sample was clipped and fixed in a sample tube containing 4% paraformaldehyde while another part was preserved in a cryostorage tube. The specific measurement indicators and methods are outlined as follows:

Determination of fasting blood glucose and related indices of glucose metabolism: The fasting blood glucose levels of diabetic and normal mice were assessed by collecting blood samples from the tail vein. Prior to measuring fasting blood glucose, the mice underwent a 12‐h overnight fast with ad libitum access to water. Following a 4‐week administration period, serum insulin (INS), glucagon‐like peptide 1 (GLP‐1), glucose‐dependent insulinotropic polypeptide (GIP), leptin, and dipeptidyl peptidase IV (DPP‐4) were quantified in all mice following the instructions provided with the kit.

Oral Glucose Tolerance Test (OGTT): After a 4‐week administration period, both the diabetic and normal groups of mice underwent a 12‐h fasting period prior to an oral glucose tolerance test (OGTT), during which they had unrestricted access to water. The glucose levels of both groups were measured at 0, 30, 60, 90, and 120 min after intragastric administration of a starch solution with a dosage of 2 mg/kg. Subsequently, an OGTT curve was plotted, and the area under the curve (AUC) was calculated [74].

Determination of serum biochemical indexes: The levels of triglyceride (TG), cholesterol (TC), high‐density lipoprotein (HDL‐C), low‐density lipoprotein (LDL‐C), alanine aminotransferase (ALT), aspartate aminotransferase (AST), glycated serum protein (GSP), LPS, and malondialdehyde (MDA) were quantified using the Nanjing‐built kit. Additionally, catalase activity (CAT), reduced glutathione levels (GSH), and superoxide dismutase activity(T‐SOD) were also measured. Please refer to the kit instructions for further details.

Determination of liver biochemical indexes: The levels of TG, TC, MDA, CAT, GSH, and SOD in the mouse liver were quantified following the instructions provided by the kit.

Histopathological observation: The liver, pancreas, spleen, and kidney tissues were fixed in a 4% paraformaldehyde solution for over 24 h. The tissues were trimmed, cut, placed in a dehydration box, and sequentially dehydrated with alcohol ranging from 75% to 100%. Subsequently, the tissue was immersed in wax and processed using an embedding machine. After dressing, the tissue was sliced using a paraffin slicing machine. The resulting sections were affixed onto slides and dried for further use. These sections underwent HE staining following the subsequent main steps: (1) treatment with methanol and xylene followed by rinsing with distilled water; (2) sequential staining with Harri hematoxylin, differentiation using 1% hydrochloric acid alcohol followed by blue restoration with 0.6% ammonia water while being washed periodically with distilled water; (3) eosin staining lasting for approximately 3–5 min; (4) dehydration and transparency achieved through different concentrations of alcohol and xylene solutions before sealing them with glue; (5) observation under a microscope accompanied by photography.

### 4.20 Stool Collection and Intestinal Microbial Analysis of Mice

After 4 weeks of intragastric administration, feces from 5 groups of mice were collected in a SPF environment, transferred to cryostorage tubes, and rapidly stored in liquid nitrogen[73]. Bacterial DNA genomes were extracted from the mouse feces using the DNA stool kit protocol, and the V3‐V4 region of the bacterial 16S rRNA genome was amplified using the following primers: 338F‐ACTCCTACGGGAGGCAGCAG; 8068‐GGACTACHVGGGTWTCTAAT. The PCR products were initially purified through 2% gel electrophoresis to obtain target bands, further purified with AxyPrep DNA Gel Extraction Kit, and finally quantified using fluorTM pico green dsDNA Kit for PCR product quantification. Purification was performed according to Illumina MiSeq platform instructions. The NA amplicon was sequenced and filtered using QIIME (version 1.17), followed by data extraction and merging with FLASH software. An operational classification unit clustering analysis was conducted on all samples based on a sequence similarity threshold of 97%. Finally, species information and abundance distribution were obtained by comparing against a database.

### 4.21 Statistical Analysis

Unless otherwise stated, the experiments were triple‐replicated, and the data were expressed as the mean ± standard deviation of the triple‐replicates. The histogram was plotted using GraphPad Prism 9.0 (GraphPad, CA, USA) and Origin 8.0 (Origin Lab Inc., MA, USA). Univariate ANOVA was performed using GraphPad Prism 9.0 (GraphPad, CA, USA) to analyze statistical significance at 95% confidence level.

## Author Contributions

F.G., L.Z., and M.W. performed the experiments and drafted the original manuscript. Y.D., T.Z., and H.C. assisted with sample preparation and data collection. J.S., Y.Y., and Z.H. contributed to formal analysis and visualization. J.A., X.Z., and W.L. provided technical support and resources. F.R. offered supervision and validated the methodology. P.W. and P.L. supervised the study, acquired funding, and reviewed and edited the manuscript. All authors have read and agreed to the published version of the manuscript.

## Conflicts of Interest

The authors declare no conflicts of interest.

## Supporting information




**Supporting File**: advs75869‐sup‐0001‐SuppMat.pdf.

## Data Availability

The data that support the findings of this study are available from the corresponding author upon reasonable request.
