# Supplementary material for: SuFEx‐Enabled Reprogramming of Flavonoids for Selective α‐Glucosidase Covalent Inhibition
Source: Adv Sci (Weinh). 2026 May 29:e75869. Online ahead of print. doi: 10.1002/advs.75869 (PMC13335957; doi:10.1002/advs.75869)
Supplement: Supplementary file 1 — Supporting File: advs75869‐sup‐0001‐SuppMat.pdf. [file ADVS-9999-e75869-s001.pdf]

Supplementary:

SuFEx-Enabled Reprogramming of Flavonoids for Selective α-Glucosidase Covalent Inhibition

Fengyu Guo[#], Liwei Zhang[#], Minlong Wang[#], Yanjin Du, Tianyu Zhang, Hao Chen, Jiazeng Sun, Yang Yu, Zude He, Jie An, Xiaoxu Zhang, Weilin Lin, Fazheng Ren, Pengjie Wang[*], Ping Liu[*]

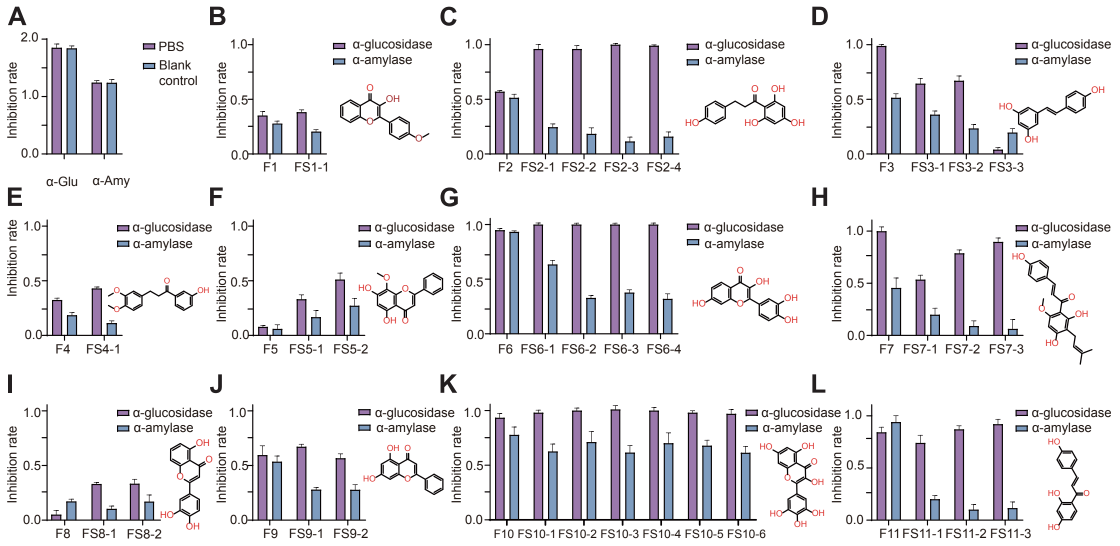

Figure S1. Enzyme inhibitory activity of arylfluorosulfates derivatives. (A) shows the effect of solvent on enzyme inhibitory activity. (B-L) represent the enzyme inhibitory activities of the compounds after derivatization of compounds F1-F10, respectively.

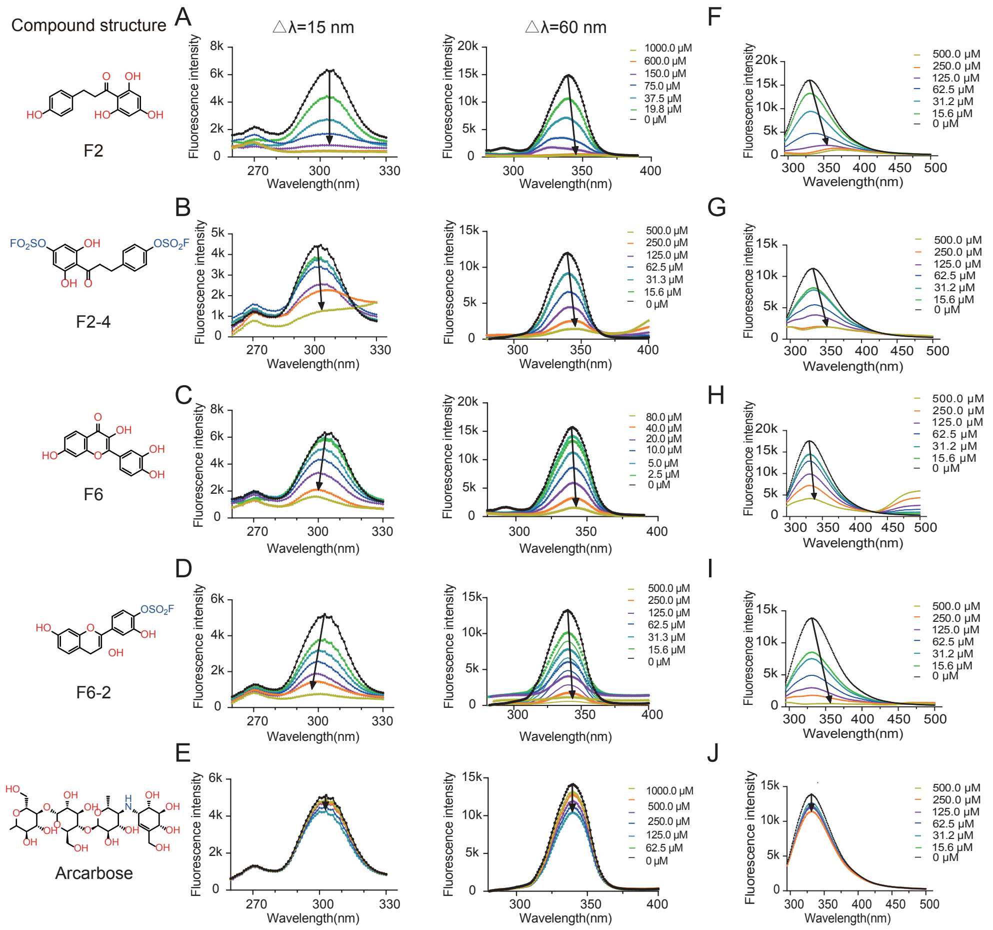

Figure S2. Degree of affinity of arylfluorosulfates derivatives for α-glucosidase. (A-E) Simultaneous fluorescence spectra of α-glucosidase at Δλ = 15 and Δλ = 60 nm by F2 (A), FS2-4 (B), F6 (C), FS6-2 (D), and Acarbose (E). (F-J) Fluorescent quenching curve of α-glucosidase by F2 (F), FS2-4 (G), F6 (H), FS6-2 (I) and Acarbose (J).

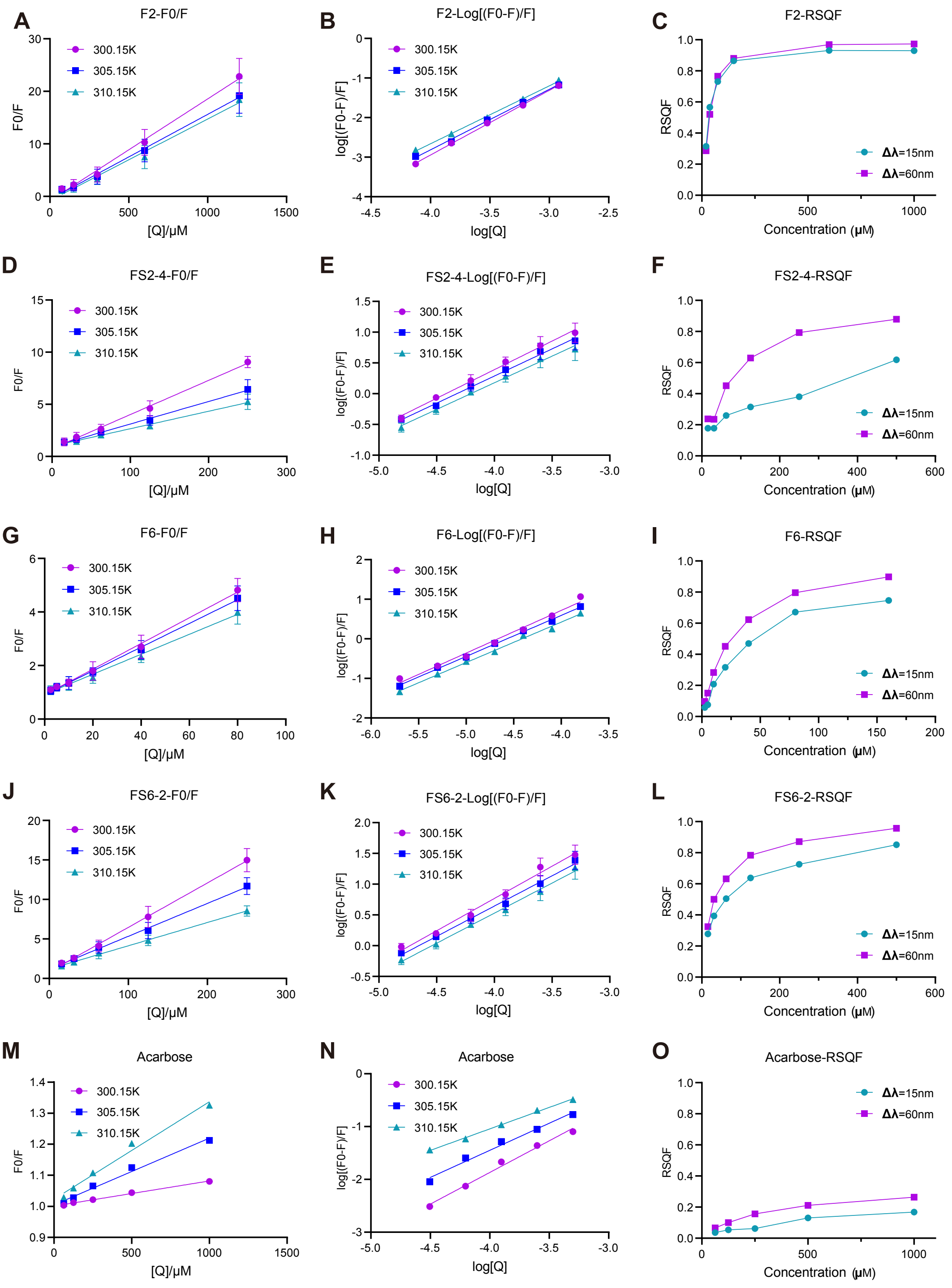

Figure S3. Fluorescence characterization of the binding affinity between enzyme and inhibitor. Fluorescence characterization of the binding affinity between enzyme and inhibitor. Stern-Volmer curve of α-glucosidase by F2 (A and B), FS2-4 (D and E), F6 (G and H), FS6-2 (J and K) and Acarbose (M and N). followed by corresponding RSFQ diagrams F2 (C), FS2-4 (F), F6 (I), FS6-2 (L), and Acarbose (O).

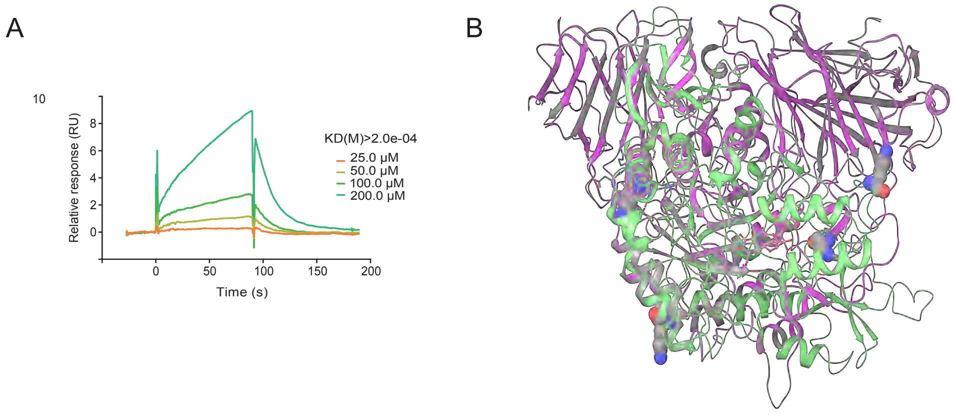

Figure S4. (A) SPR interaction results between α-glucosidase Lys 480 amino acid residue mutant and FS6-2. (B) Structural superimposition results of human-derived α-glucosidase (green) and yeast-derived α-glucosidase (purple).

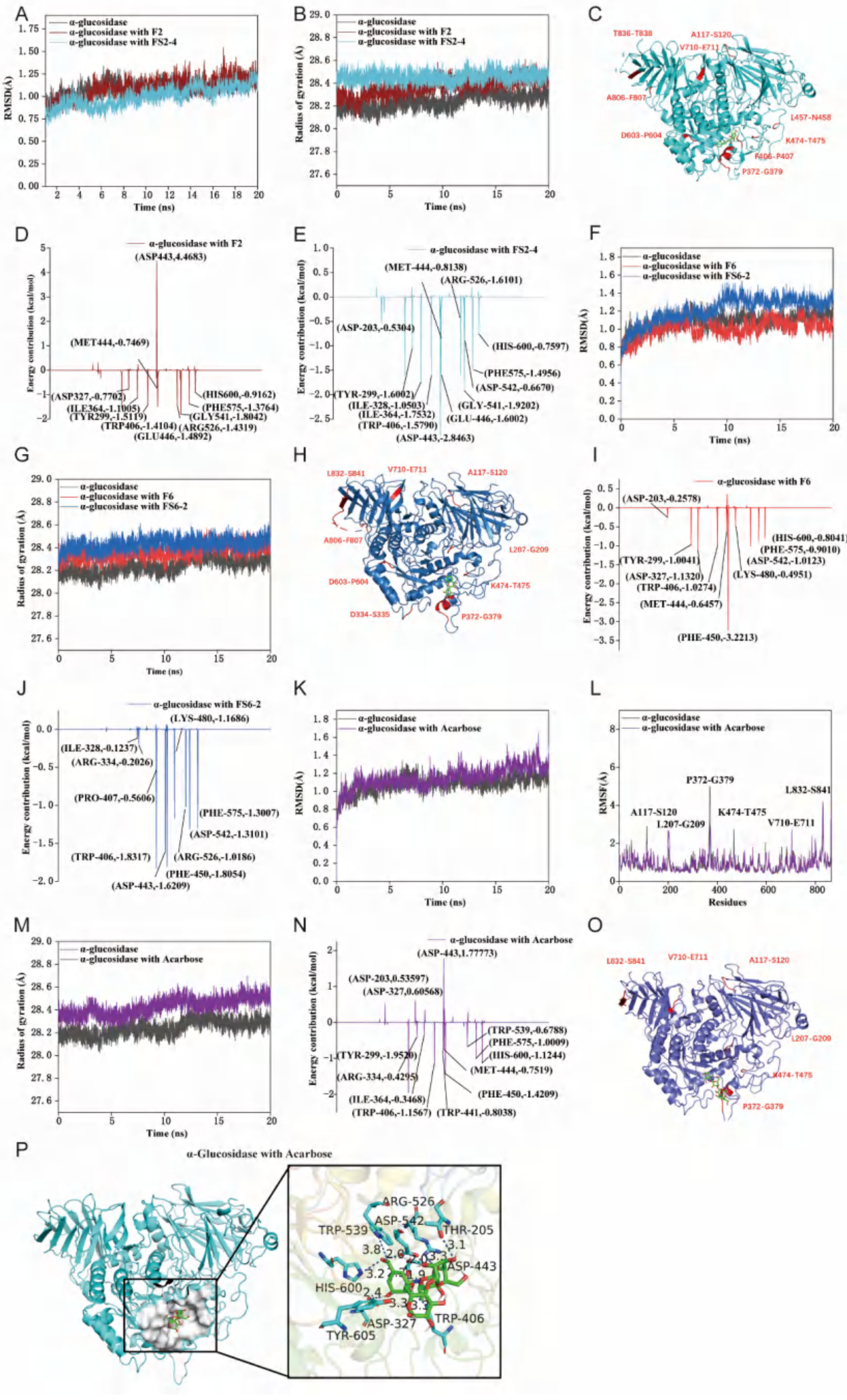

A
α-glucosidase
α-glucosidase with F2
α-glucosidase with FS2-4
RMSD(Å)
Time (ns)
B
Radius of gyration (Å)
C
T836-T838
A117-S120
V710-E711
A806-F807
L457-N458
D603-P604
K474-T475
F406-P407
P372-G379
D
α-glucosidase with F2
(ASP443,4.4683)
(MET444,-0.7469)
(ASP327,-0.7702)
(ILE364,-1.1005)
(TYR299,-1.5119)
(TRP406,-1.4104)
(GLU446,-1.4892)
(HIS600,-0.9162)
(PHE575,-1.3764)
(GLY541,-1.8042)
(ARG526,-1.4319)
Energy contribution (kcal/mol)
E
α-glucosidase with FS2-4
(MET-444,-0.8138)
(ARG-526,-1.6101)
(ASP-203,-0.5304)
(HIS-600,-0.7597)
(PHE575,-1.4956)
(ASP-542,-0.6670)
(TYR-299,-1.6002)
(ILE-328,-1.0503)
(ILE-364,-1.7532)
(TRP-406,-1.5790)
(GLY-541,-1.9202)
(GLU-446,-1.6002)
(ASP-443,-2.8463)
F
α-glucosidase
α-glucosidase with F6
α-glucosidase with FS6-2
G
H
L832-S841
V710-E711
A117-S120
A806-F807
L207-G209
D603-P604
K474-T475
D334-S335
P372-G379
I
α-glucosidase with F6
(ASP-203,-0.2578)
(HIS-600,-0.8041)
(PHE-575,-0.9010)
(ASP-542,-1.0123)
(TYR-299,-1.0041)
(ASP-327,-1.1320)
(TRP-406,-1.0274)
(LYS-480,-0.4951)
(MET-444,-0.6457)
(PHE-450,-3.2213)
J
α-glucosidase with FS6-2
(LYS-480,-1.1686)
(ILE-328,-0.1237)
(ARG-334,-0.2026)
(PRO-407,-0.5606)
(PHE-575,-1.3007)
(ASP-542,-1.3101)
(TRP-406,-1.8317)
(ARG-526,-1.0186)
(PHE-450,-1.8054)
(ASP-443,-1.6209)
K
α-glucosidase
α-glucosidase with Acarbose
L
RMSF(Å)
Residues
A117-S120
L207-G209
P372-G379
K474-T475
V710-E711
L832-S841
M
N
α-glucosidase with Acarbose
(ASP-443,1.77773)
(ASP-203,0.53597)
(ASP-327,0.60568)
(TRP-539,-0.6788)
(PHE-575,-1.0009)
(HIS-600,-1.1244)
(TYR-299,-1.9520)
(MET-444,-0.7519)
(ARG-334,-0.4295)
(PHE-450,-1.4209)
(ILE-364,-0.3468)
(TRP-406,-1.1567)
(TRP-441,-0.8038)
O
L832-S841
V710-E711
A117-S120
L207-G209
K474-T475
P372-G379
P
α-Glucosidase with Acarbose
ARG-526
ASP-542
THR-205
TRP-539
ASP-443
HIS-600
TRP-406
TYR-605
ASP-327

Figure S5. Molecular dynamics simulations of inhibitors with α-glucosidase. (A), (F) and (K) depict the dynamic changes in RMSD curves for the protein complex and free protein systems over a 20 ns simulation period. (B), (G) and (M) represent the rotation radii of the α-glucosidase system under complexation conditions compared to those of the free α-glucosidase system. (C), (H) and (O) illustrate the structures of α-glucosidase, with regions exhibiting high fluctuations highlighted in red. The contribution of residual energy was analyzed during molecular dynamics simulations of α-glucosidase complexed with compounds F2(D), FS2-4(E), F6(I), FS6-2(J), and Acarbose (N). (L) present the RMSF values of binding site residues in both free proteins and complex systems. (P) Molecular docking of α-glucosidase with Acarbose. The dashed blue lines represent hydrogen bonds, the dashed gray lines represent hydrophobic interactions, the dashed green lines represent π-π stacking, and the dashed yellow lines represent salt bridge (lonic bond).

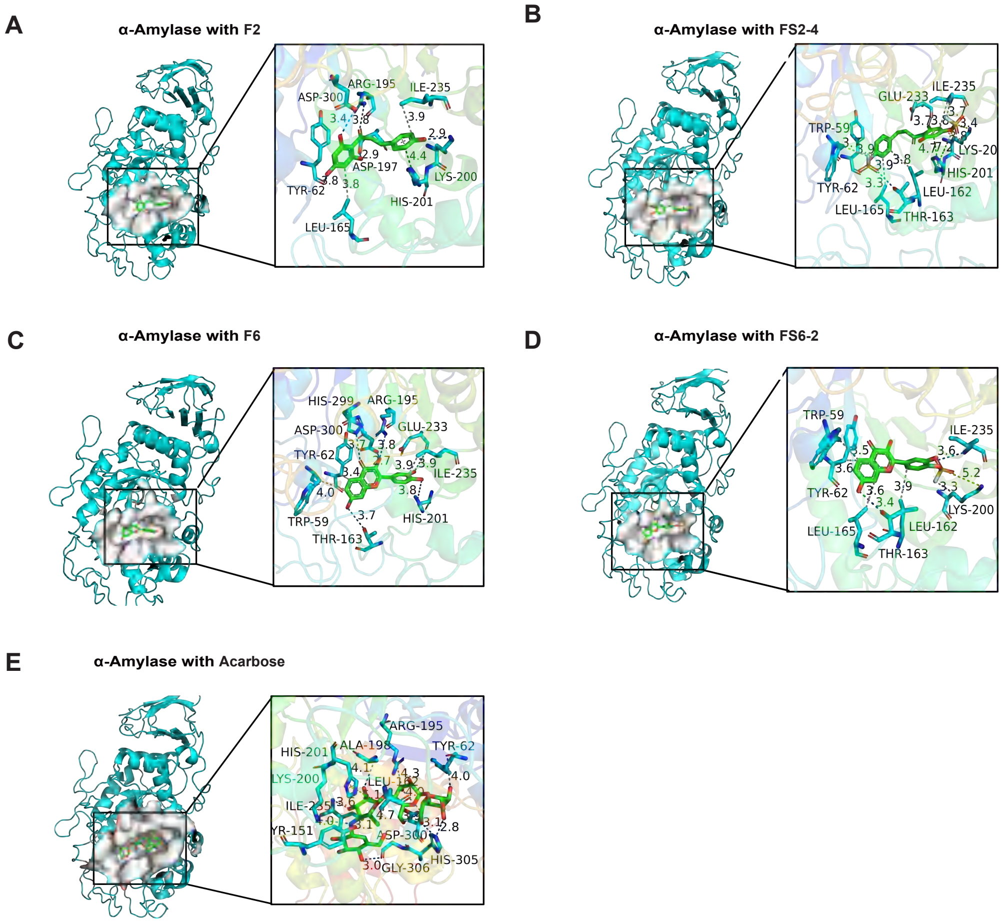

Figure S6. Molecular docking of α-amylase with F2 (A), FS2-4 (B), F6 (C), FS6-2 (D), and Acarbose (E). The dashed blue lines represent hydrogen bonds, the dashed gray lines represent hydrophobic interactions, the dashed green lines represent π-π stacking, and the dashed yellow lines represent salt bridge (Ionic bond).

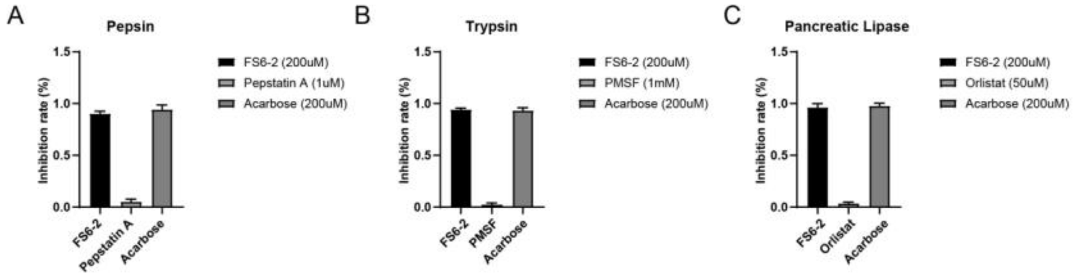

Figure S7. Inhibitory effect of FS6-2 on representative digestive enzymes in the gastrointestinal tract. (A) Inhibitory effects of FS6-2 and acarbose on pepsin. (B) Inhibitory effects of FS6-2 and acarbose on trypsin. (C) Inhibitory effects of FS6-2 and acarbose on pancreatic lipase.

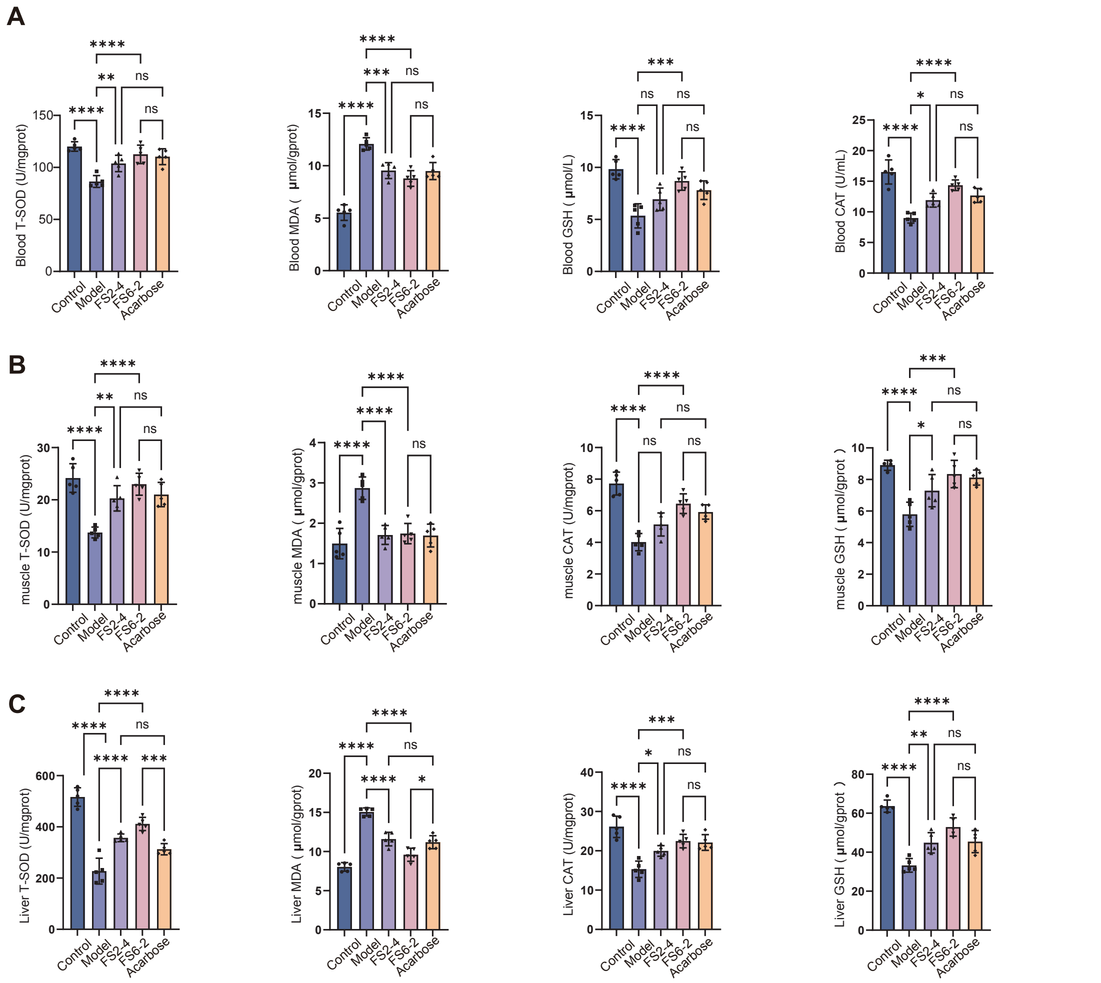

Figure S8. Effect of inhibitors on antioxidant capacity in dbdb mice. (A) represent the blood levels of MDA, GSH, CAT and T-SOD in dbdb mice, respectively. (B) represent the liver levels of MDA, GSH, CAT and T-SOD in dbdb mice, respectively. (C) represent the muscle levels of MDA, GSH, CAT and T-SOD in dbdb mice, respectively.

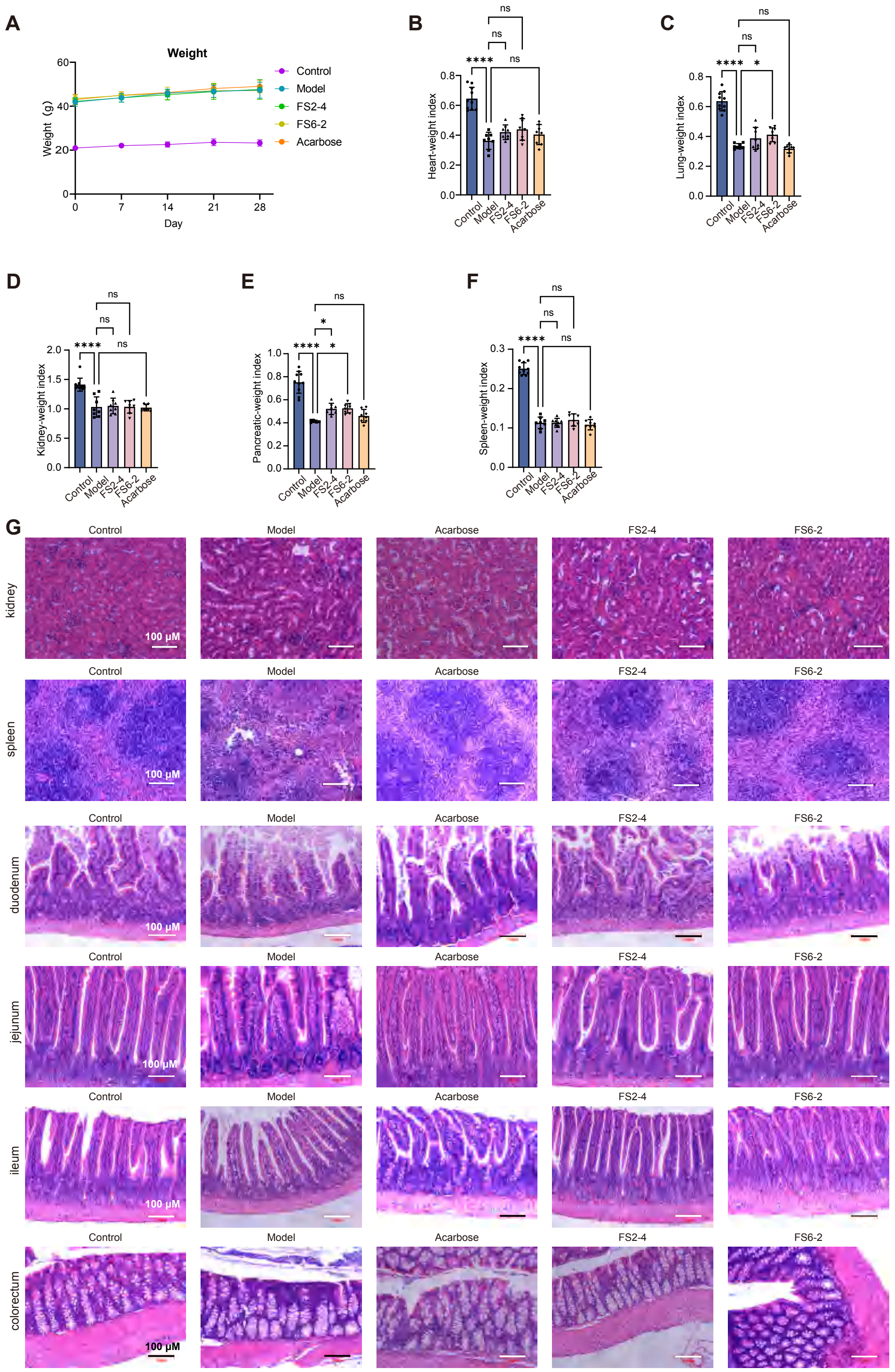

Figure S9. Organ indices as well as blood biochemical indices after inhibitors treatment. (A) Body weight of experimental mice administered for 28 days. (B-F) Organ indices corresponding to heart, lung, kidney, pancreas, and spleen, respectively. (G) The HE staining results of kidney, spleen, duodenum, jejunum, ileum, and colorectum tissues.

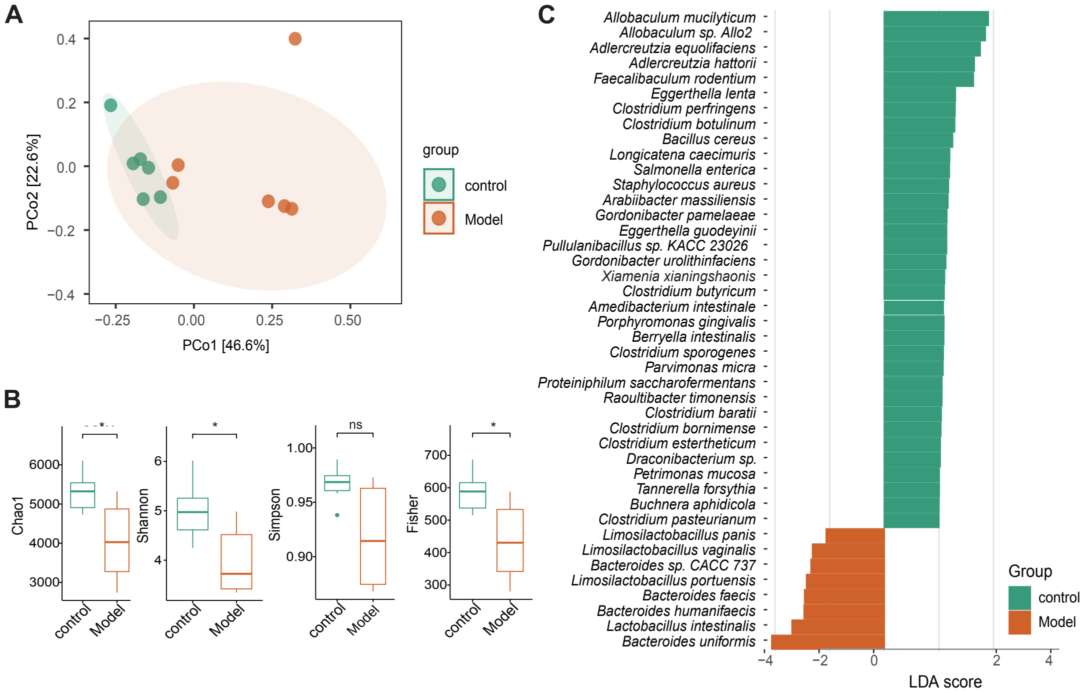

Figure S10. Analysis of molding results. (A) is the principal component analysis plot of control and model groups. (B) represent the differences between control and model groups calculated using chao1, Shannon, Simpson and Fisher methods, respectively. (C) is the plot of LDA scores of control and model groups.

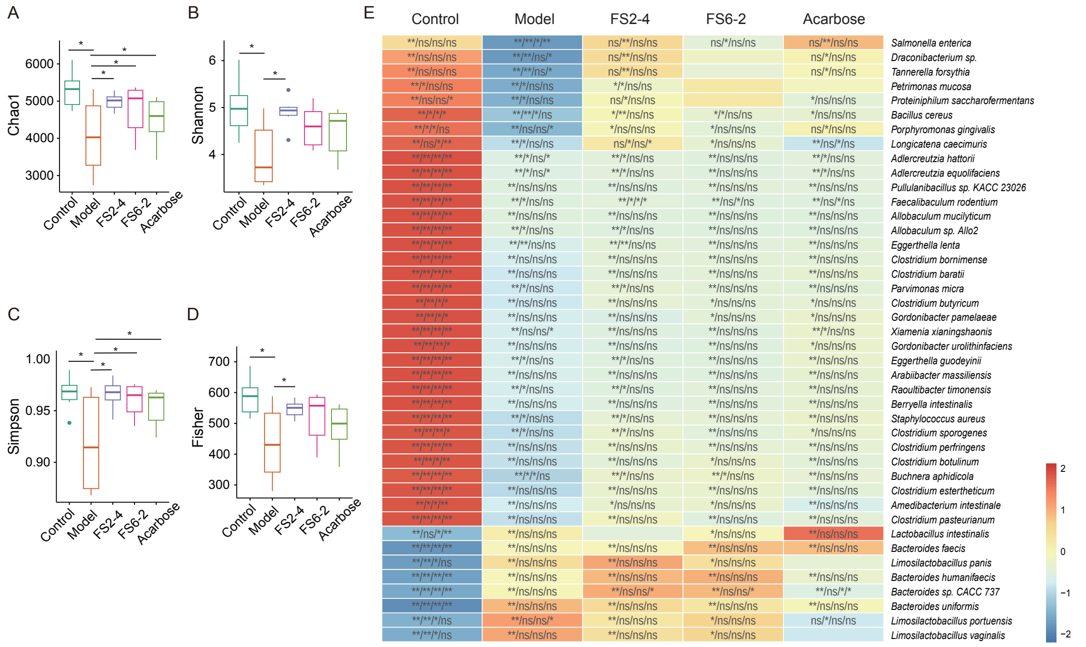

Figure S11. Analysis of differences across intervention groups. (A-D) represent the differences in control, model, FS2-4, FS6-2, and acarbose groups calculated using the chao1, Shannon, Simpson, and Fisher methods, respectively. (E) Differential strains induced by modeling in each group, degree of recovery after intervention.

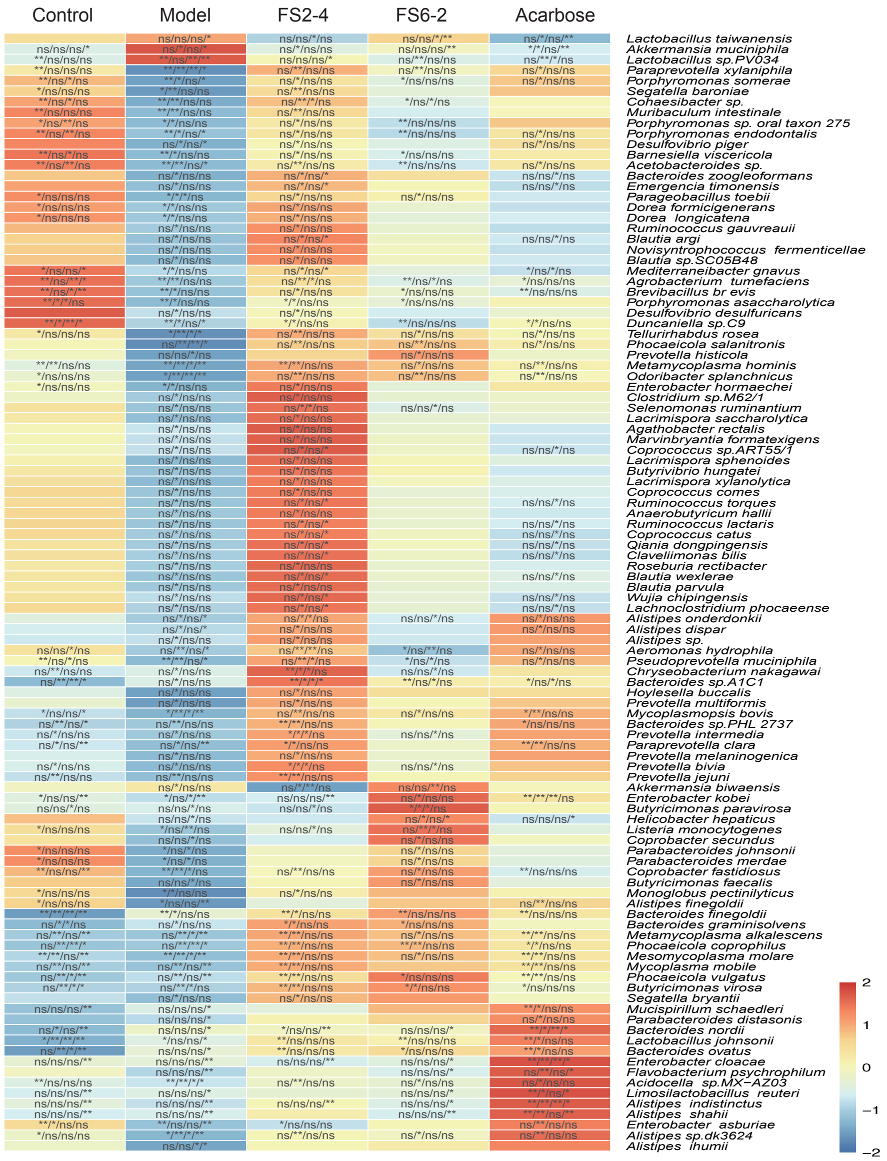

Figure S12. Differential strains induced by modeling, abundance statistics of 106 differential bacteria between intervention and model groups.

Table S1. Flavonoid structure and coding for SuFEx click chemistry

ID number	structure	CAS number
F1	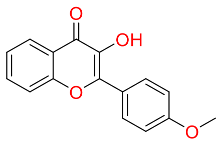	6889-78-7
F2	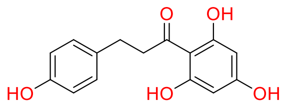	60-82-2
F3	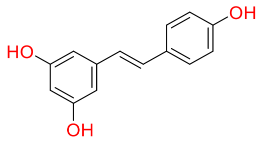	501-36-0
F4	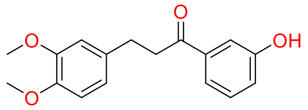	178445-83-5
F5	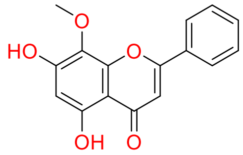	632-85-9
F6	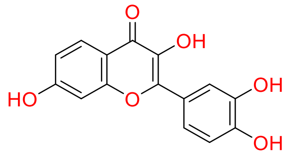	1174865-69-0
F7	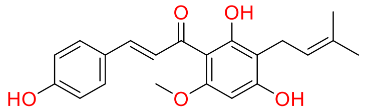	6754-58-1
F8	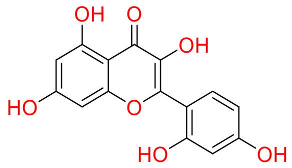	480-16-0
F9	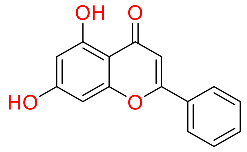	480-40-0
F10	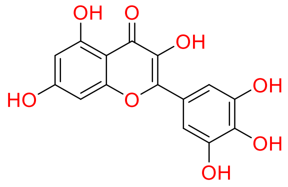	529-44-2

F11	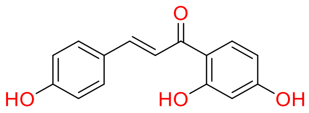	961-29-5

Table S2. Kinetic parameters of α-glucosidase in the absence and presence of inhibitors.

Compound	[I] (μM)	Type of inhibition	V_{max} (×10^{-1}μM/min)	K_m (μM)	K_i (μM)	K_{ic} (μM)	K_{iu} (μM)
-	0	-	5.95±0.02	5.83±0.12	-	-	-
F2	120	Competitive	6.07±0.02	9.56±1.23	182.6±5.9	108.07±3.87	-
	100		6.09±0.05	9.01±0.99			
	80		6.20±0.03	8.48±0.68			
	60		6.18±0.06	7.81±1.77			
	40		6.19±0.04	7.22±1.57			
FS2-4	500	Competitive	6.07±0.04	18.12±1.59	56.92±15.21	53.67±11.57	-
	250		6.12±0.03	28.87±1.98			
	125		6.03±0.04	10.42±1.05			
	62.5		6.07±0.02	6.57±1.07			
	31.25		6.11±0.06	5.59±0.51			
	15.63		6.10±0.05	6.45±2.06			
F6	80	Competitive	5.76±0.03	31.85±2.43	26.69±7.77	13.44±2.68	-
	62.5		6.05±0.07	20.90±1.37			
	31.25		6.21±0.06	13.28±0.92			
	15.63		5.46±0.08	8.12±0.69			
FS6-2	250	Competitive	6.20±0.06	60.71±4.45	9.24±1.87	6.04±2.87	-
	125		6.09±0.04	30.90±3.93			
	62.5		6.24±0.02	13.07±2.06			
	31.25		6.03±0.08	7.53±1.34			
Acarbose	2000	Mixed	6.06±0.06	29.36±4.81	574.13±57.2	556.17±19.16	540.94±49.36
	1000		5.90±0.03	18.84±2.95			
	500		6.18±0.04	13.32±3.28			
	250		5.92±0.06	9.58±2.29			
	125		5.96±0.06	7.47±0.95			

Table S3. Quenching constants (Ksv), binding constants (Ka), and thermodynamic parameters of inhibitors with α-glucosidase interaction at different temperatures.

System	T (K)	K_{sv} (10^4 /M)	K_q $(M·S)^{-1}$	R^2	*n*	K_a (10^4 /M)	R^2
	300.15	1.94 ±0.08	$1.94×10^{12}$	0.99	1.15	0.38 ±1.23	0.99
F2	305.15	1.62 ±0.06	$1.62×10^{12}$	0.99	1.09	0.20 ±0.24	0.99
	310.15	1.55 ±0.01	$1.55×10^{12}$	0.98	1.01	0.16 ±0.06	0.99
	300.15	3.27 ±0.01	$3.27×10^{12}$	0.99	0.93	1.28 ±0.02	0.99
FS2-4	305.15	2.16 ±0.01	$2.16×10^{12}$	0.99	0.99	0.65 ±0.01	0.99
	310.15	1.67 ±0.01	$1.67×10^{12}$	0.99	0.99	0.44 ±0.01	0.99
	300.15	4.80 ±0.01	$4.80×10^{12}$	0.99	1.07	9.20 ±1.23	0.99
F6	305.15	4.16±0.01	$4.42×10^{12}$	0.99	1.03	3.80±0.01	0.99
	310.15	2.95 ±0.01	$3.73×10^{12}$	0.99	1.02	2.75±0.01	0.99
	300.15	5.62 ±0.01	$5.62×10^{12}$	0.99	1.04	10.54 ±0.01	0.98
FS6-2	305.15	4.42 ±0.01	$4.16×10^{12}$	0.99	0.98	5.43 ±0.24	0.99
	310.15	3.73±0.01	$2.95×10^{12}$	0.99	0.98	3.20 ±0.06	0.99
	300.15	0.01 ±0.01	$0.01×10^{12}$	0.99	1.04	0.08 ±1.23	0.98
Acarbose	305.15	0.02 ±0.01	$0.02×10^{12}$	0.99	0.98	0.04 ±0.24	0.99
	310.15	0.03 ±0.01	$0.03×10^{12}$	0.99	0.98	0.02 ±0.06	0.99

Each value is the mean ± SEM from at least three independent experiments. The different superscripts in the same column mean significant differences ($P < 0.05$).

Table S4. The thermodynamic parameters ΔH, ΔS, and ΔG of inhibitors with α-glucosidase and α-amylase interaction at different temperatures.

System	T (K)	ΔH (kJ/mol)	ΔG (KJ/mol)	ΔS (J/mol/K)
	300.15		-20.47 ±0.29	
F2	305.15	-70.94 ±18.17	-19.63 ±0.34	-168.15 ±59.58
	310.15		-18.79 ±00.30	
	300.15		-23.50 ±0.18	
FS2-4	305.15	-83.10 ±11.89	-22.51 ±0.51	-198.56 ±36.67
	310.15		-21.52 ±0.19	
	300.15		-27.27 ±0.38	
F6	305.15	-91.89±4.95	-27.18 ±0.28	-210.24±16.24
	310.15		-26.10 ±0.39	
	300.15		-28.79 ±0.78	
FS6-2	305.15	-93.32±13.87	-27.73 ±0.26	-216.75±18.25
	310.15		-26.68 ±0.83	
	300.15		-15.72 ±0.13	
Acarbose	305.15	-38.02±8.08	-15.35 ±0.24	-74.31±2.64
	310.15		-14.98 ±0.13	

Each value is the mean ± SEM from at least three independent experiments. The different superscripts in the same column mean significant differences ($P < 0.05$).

Table S5. Molecular docking scores of inhibitors and acarbose with α-glucosidase.

Ligand with enzyme	Grid Score(kcal/mol)	Grid_vdw(kcal/mol)	Grid_es(kcal/mol)
F2	-55.09	-44.46	-10.62
FS2-4	-56.66	-43.47	-13.18
F6	-62.05	-43.55	-18.51
FS6-2	-70.15	-50.64	-19.51
Acarbose	-53.45	-41.35	-12.10

Table S6. Molecular docking scores of inhibitors and acarbose with α-amylase.

Ligand with enzyme	Grid Score(kcal/mol)	Grid_vdw(kcal/mol)	Grid_es(kcal/mol)
F2	-43.27	-39.39	-3.87
FS2-4	-42.75	-40.59	-2.16
F6	-51.40	-44.91	-6.49
FS6-2	-40.97	-37.15	-3.82
Acarbose	-53.45	-41.35	-12.10

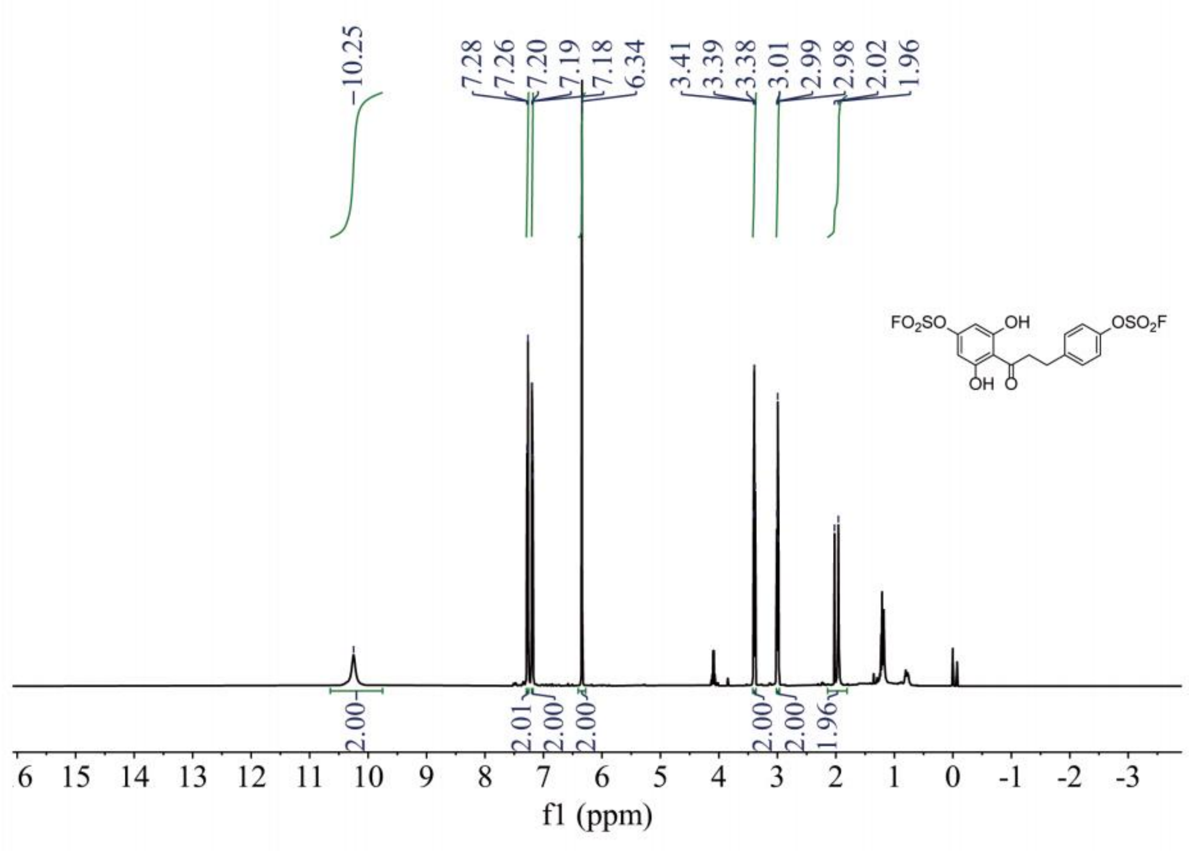

Figure S13. ^{1}HNMR verification of FS2-4. ^{1}H NMR (500 MHz, CDCl3) δ 10.25 (s, 2H), 7.27 (d, J = 8.9 Hz, 2H), 7.20 – 7.18 (m, 2H), 6.34 (s, 2H), 3.39 (t, J = 7.5 Hz, 2H), 2.99 (t, J = 7.5 Hz, 2H), 1.99 (d, J = 33.1 Hz, 2H).

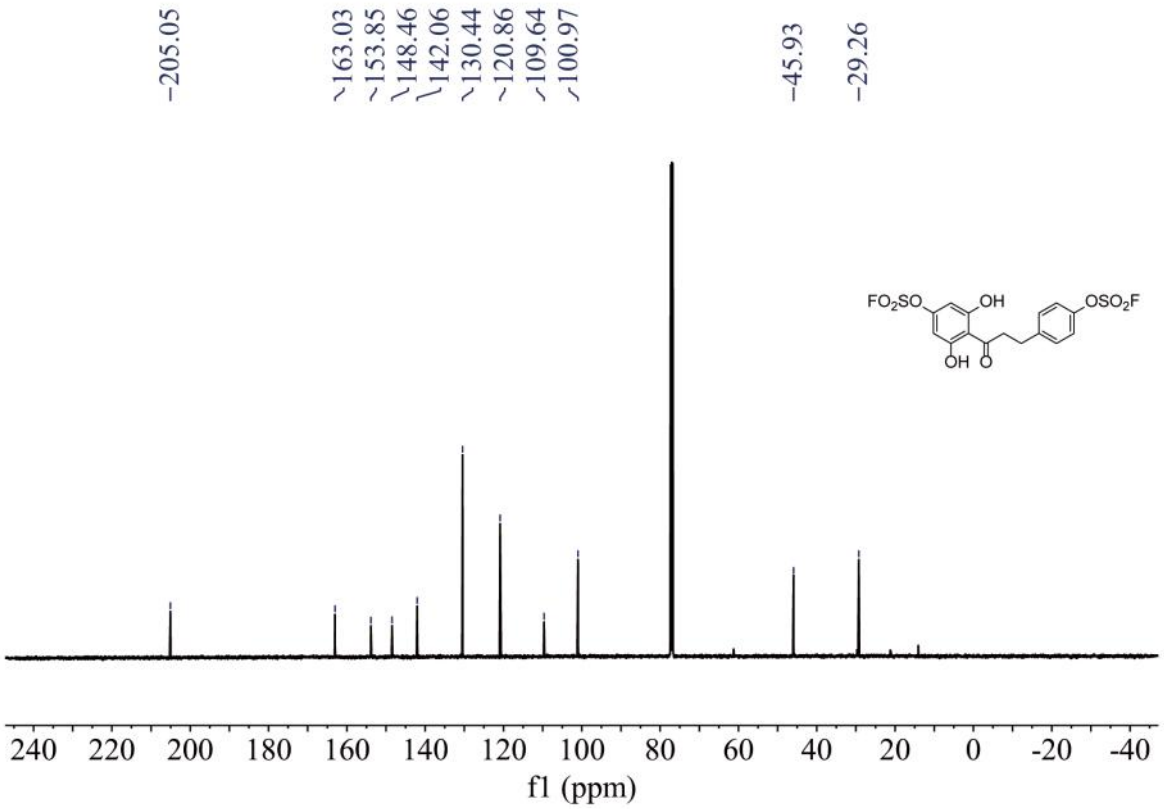

FigureS14. ^{13}CNMR verification of FS2-4. ^{13}C NMR (126 MHz, CDCl3) δ 205.05, 163.03, 153.85, 148.46, 142.06, 130.44, 120.86, 109.64, 100.97, 45.93, 29.26.

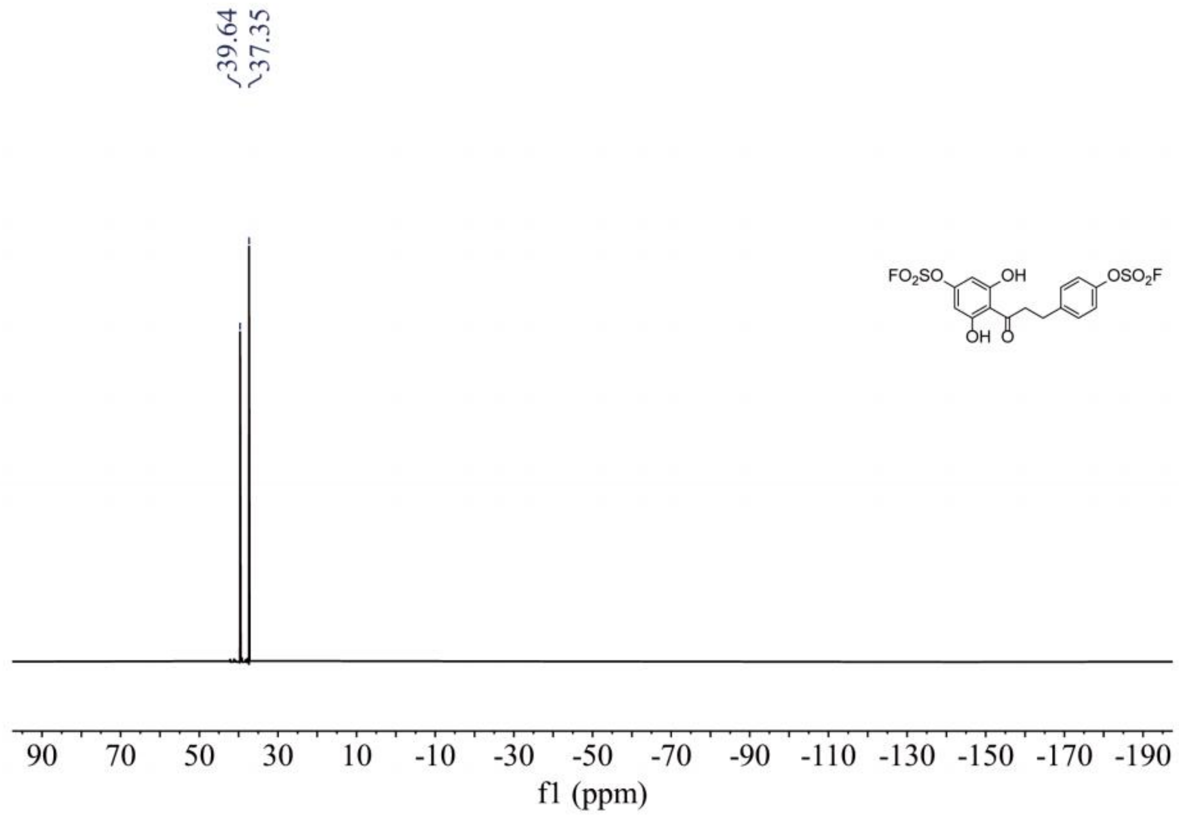

Figure S15. ^{19}FNMR verification of FS2-4. ^{19}F NMR (471 MHz, CDCl3) δ 39.64, 37.35.

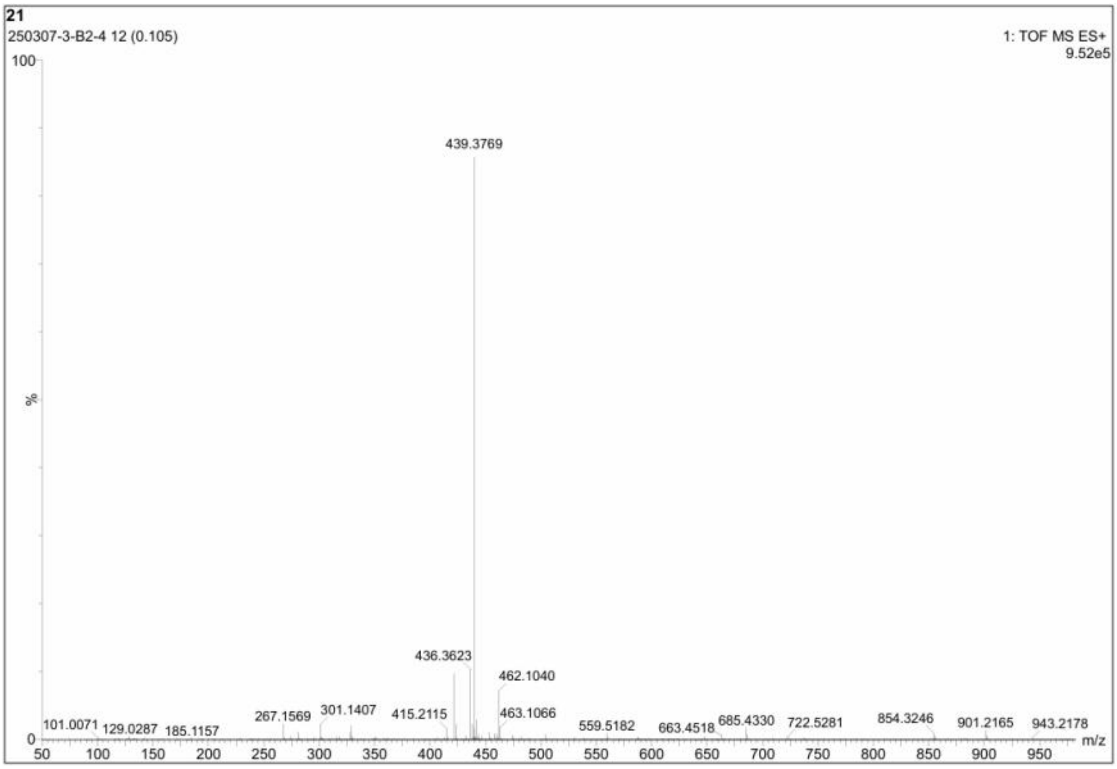

Figure S16. HRMS verification of FS2-4.

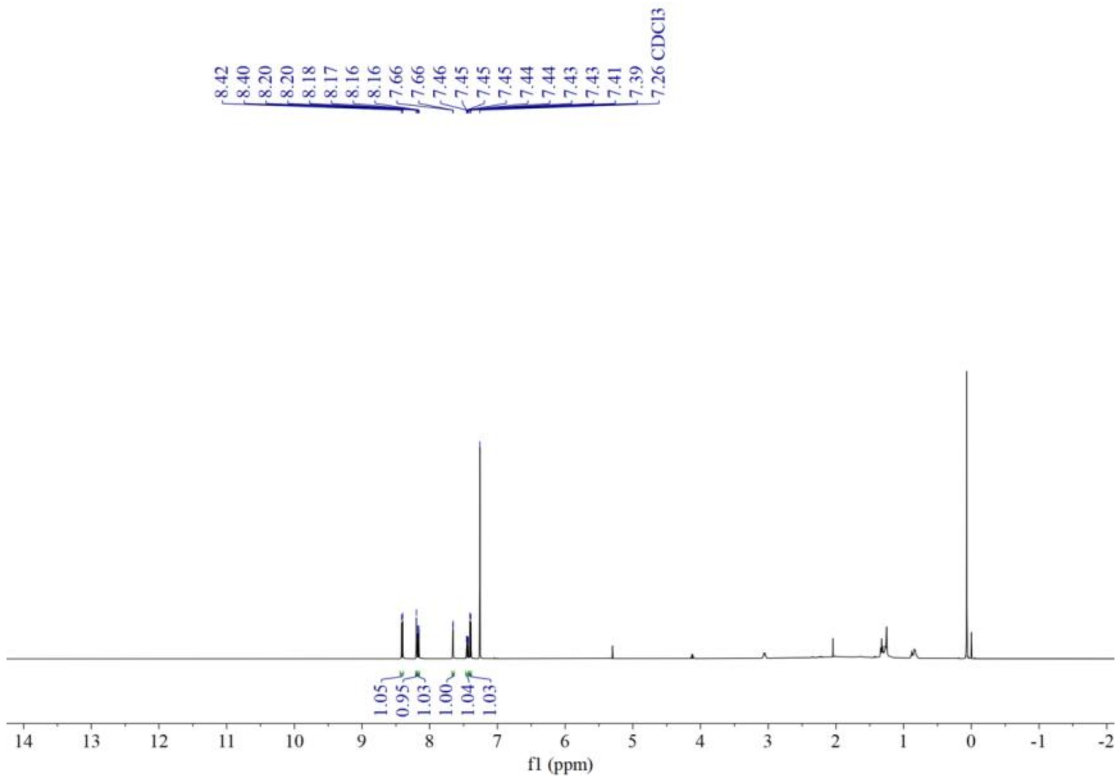

Figure S17. ^{1}HNMR verification of FS6-2. ^{1}H NMR (500 MHz, CDCl3) δ 8.41 (d, J = 8.9 Hz, 1H), 8.20 (d, J = 1.8 Hz, 1H), 8.17 (dd, J = 8.7, 1.8 Hz, 1H), 7.66 (d, J = 2.3 Hz, 1H), 7.44 (dd, J = 9.4, 2.7 Hz, 1H), 7.40 (d, J = 8.7 Hz, 1H).

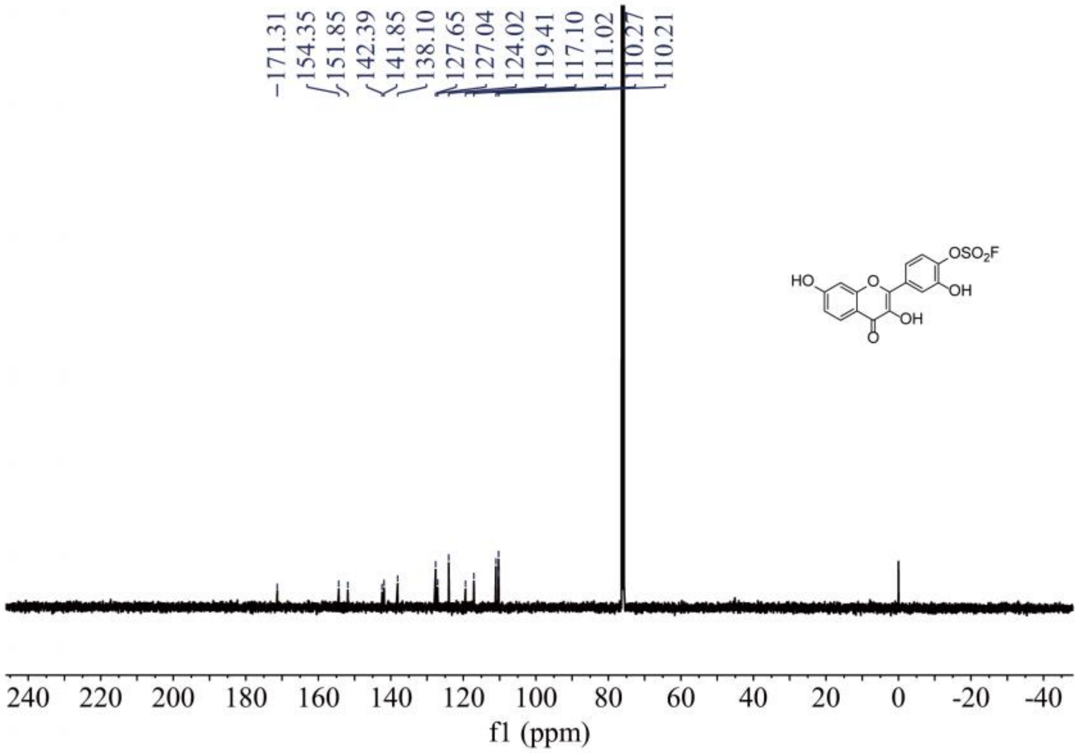

Figure S18. ^{13}CNMR verification of FS6-2. ^{13}C NMR (126 MHz, CDCl3) δ 171.31, 154.35, 151.85, 142.39, 141.85, 138.10, 127.65, 124.02, 119.41, 117.10, 111.02, 110.27, 110.21.

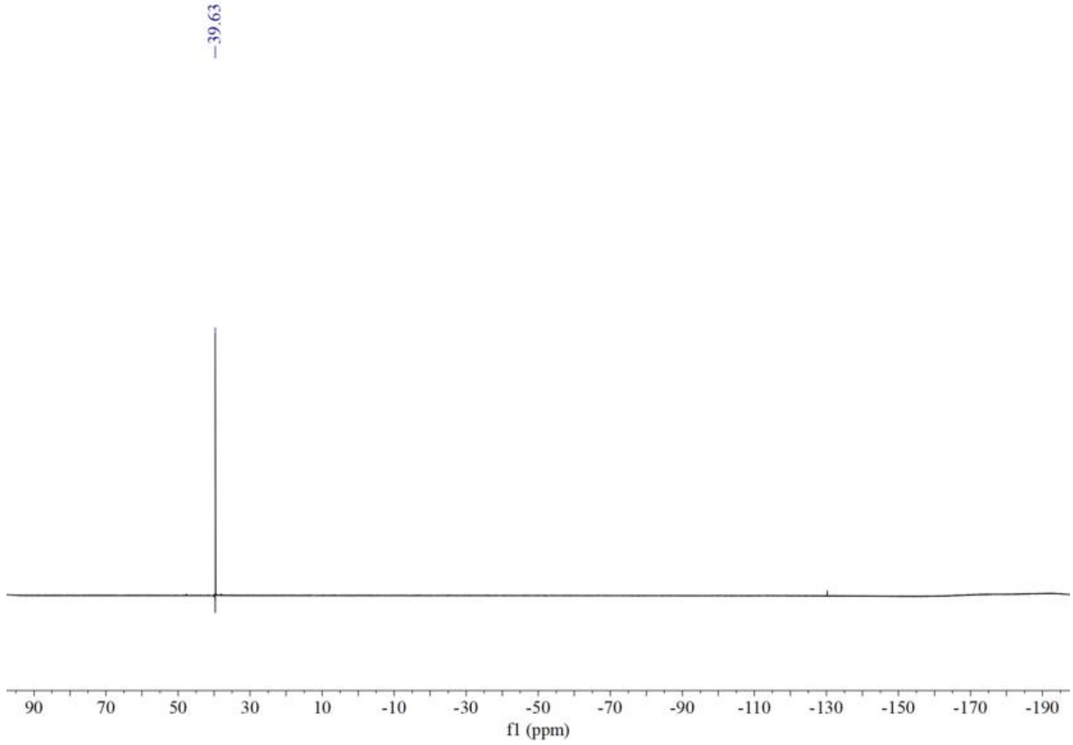

Figure S19. ^{19}FNMR verification of FS6-2. ^{19}F NMR (471 MHz, CDCl3) δ 39.63.

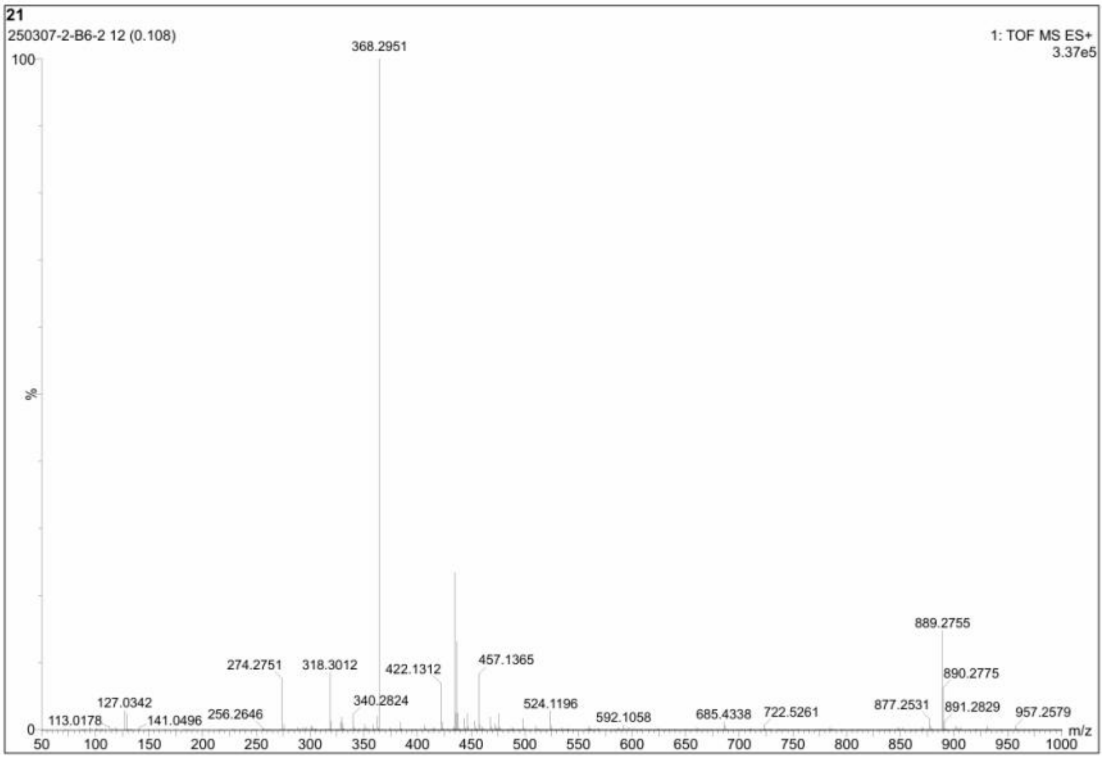

Figure S20. HRMS verification of FS6-2.

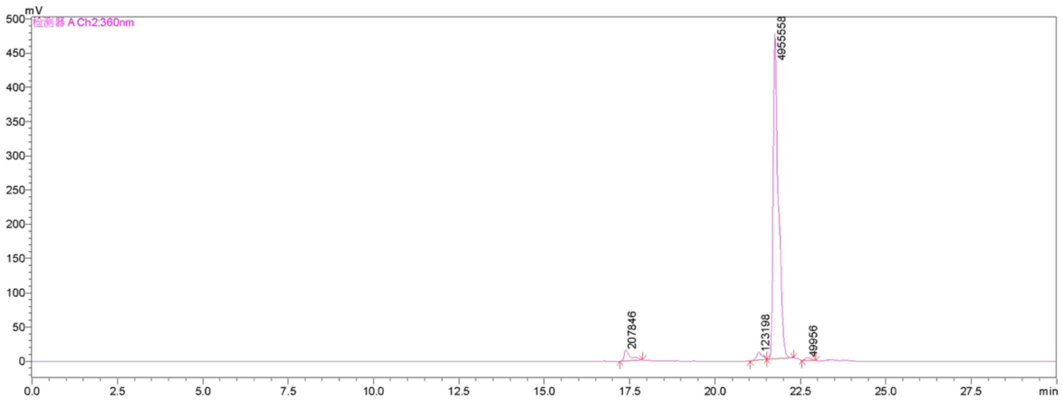

Figure S21. HPLC purity verification of FS6-2.
